# The release and trans-synaptic transmission of Tau via exosomes

**DOI:** 10.1186/s13024-016-0143-y

**Published:** 2017-01-13

**Authors:** Yipeng Wang, Varun Balaji, Senthilvelrajan Kaniyappan, Lars Krüger, Stephan Irsen, Katharina Tepper, RamReddy Chandupatla, Walter Maetzler, Anja Schneider, Eckhard Mandelkow, Eva-Maria Mandelkow

**Affiliations:** 1 German Center for Neurodegenerative Diseases (DZNE), Bonn, Germany; 2 CAESAR Research Center, Bonn, Germany; 3 Department of Neurology, University of Tübingen, Tübingen, Germany; 4 Department for Psychiatry and Psychotherapy, University Medical Center, Goettingen, Germany; 5 MPI for Experimental Medicine, Göttingen, Germany; 6 MPI for Metabolism Research, Hamburg Outstation, c/o DESY, Hamburg, Germany

**Keywords:** Tau, Alzheimer disease, Exosomes, Spreading

## Abstract

**Background:**

Tau pathology in AD spreads in a hierarchical pattern, whereby it first appears in the entorhinal cortex, then spreads to the hippocampus and later to the surrounding areas. Based on this sequential appearance, AD can be classified into six stages (“Braak stages”). The mechanisms and agents underlying the progression of Tau pathology are a matter of debate. Emerging evidence indicates that the propagation of Tau pathology may be due to the transmission of Tau protein, but the underlying pathways and Tau species are not well understood. In this study we investigated the question of Tau spreading via small extracellular vesicles called exosomes.

**Methods:**

Exosomes from different sources were analyzed by biochemical methods and electron microscopy (EM) and cryo-EM. Microfluidic devices that allow the culture of cell populations in different compartments were used to investigate the spreading of Tau.

**Results:**

We show that Tau protein is released by cultured primary neurons or by N2a cells overexpressing different Tau constructs via exosomes. Neuron-derived exosomal Tau is hypo-phosphorylated, compared with cytosolic Tau. Depolarization of neurons promotes release of Tau-containing exosomes, highlighting the importance of neuronal activity. Using microfluidic devices we show that exosomes mediate trans-neuronal transfer of Tau depending on synaptic connectivity. Tau spreading is achieved by direct transmission of exosomes between neurons. In organotypic hippocampal slices, Tau-containing exosomes in conditioned medium are taken up by neurons and microglia, not astrocytes. In N2a cells, Tau assemblies are released via exosomes. They can induce inclusions of other Tau molecules in N2a cells expressing mutant human Tau. We also studied exosomes from cerebrospinal fluid in AD and control subjects containing monomeric and oligomeric Tau. Split-luciferase complementation reveals that exosomes from CSF can promote Tau aggregation in cultured cells.

**Conclusion:**

Our study demonstrates that exosomes contribute to trans-synaptic Tau transmission, and thus offer new approches to control the spreading of pathology in AD and other tauopathies.

**Electronic supplementary material:**

The online version of this article (doi:10.1186/s13024-016-0143-y) contains supplementary material, which is available to authorized users.

## Background

Tau aggregates are one of the hallmarks of Alzheimer disease (AD) [1]. In AD, the distribution of Tau aggregates correlates with the severity of dementia and occurs in a hierarchical pattern, whereby it first appears in the entorhinal cortex, then spreads to the hippocampus and later to the surrounding areas [2]. Based on this sequential appearance, AD can be classified into 6 stages [3]. Up to date, the mechanism underlying the progression of Tau pathology remains elusive. It has become of major interest, as blocking the propagation of Tau pathology might represent a promising therapeutic strategy for AD and other tauopathies.

In AD, neurons prone to develop NFTs are projection neurons with long axons that are only poorly myelinated [4]. This led to the assumption that these neurons are intrinsically vulnerable to Tau pathology, because they have high energy requirements (that is, high metabolic rates) that may subject them to chronic oxidative stress [5]. In addition their poor myelination increases their exposure to toxic environmental conditions, as myelin sheaths provide a mechanical barrier against pathogens and provide trophic support and protection against oxidative stress [6]. On the other hand, neurons affected by Tau pathology appear to be anatomically connected [7]. Thus, it is not clear whether the spread of Tau pathology is due to their neuronal connectivity or due to the differential vulnerability of neurons to Tau pathology. Emerging evidence supports the former proposal. Several recent studies showed that inoculation of Tau aggregates induces time-dependent spreading of Tau pathology from the inoculation site to synaptically connected brain regions in human Tau transgenic mice or even in wild type mice. In two lines of Tau transgenic mice, the expression of human Tau under the control of the neuropsin promoter was restricted to the entorhinal cortex, yet Tau aggregates composed of transgenic human Tau and endogenous mouse Tau were detected in brain regions downstream in the synaptic circuit such as the dentate gyrus, CA fields of the hippocampus, and cingulate cortex [8, 9]. Since no expression of human Tau was detected in these regions, human Tau in these areas should derive from the entorhinal cortex. Taken together, these studies indicate that Tau may be transmitted between neurons by a trans-synaptic pattern. However, the mechanism of transmission remains a matter of debate.

Tau is a microtubule associated protein and as such is primarily an intracellular protein. However, several recent studies showed that Tau can be physiologically released to the extracellular fluid both in vivo and in cultured cells, and such release appears to be regulated by neuronal activity [10–13]. So far, the physiological function of extracellular Tau remains elusive. In vitro and in vivo studies showed that exogenous Tau aggregates can be internalized by neurons and act as seeds to induce the aggregation of other Tau proteins [14–17]. Based on these findings, it has been proposed that the release and uptake of Tau underlies the spreading of Tau pathology [18]. However, how and what Tau species are released by donor neurons and then taken up by recipient neurons is not clear.

Exosomes are small extracellular vesicles with a diameter of 50–150 nm, which derive from multivesicular endosomes fusing with the plasma membrane [19, 20]. Secreted exosomes can be taken up by and thus deliver their content to the recipient cells [21] thus representing a novel intercellular communication pathway. Exosomes can be released at neuronal presynaptic terminals and taken up by postsynapses at *Drosophila* neuromuscular junctions (NMJ) [22], and therefore qualify as carriers for trans-synaptic transmission of proteins. Therefore, it is reasonable to assume that exosomes might be involved in the trans-synaptic spreading of Tau pathology. It has been reported that α-synuclein, prion protein and β-amyloid are present in exosomes [23–25], but whether or not Tau is a component of exosomes is still a matter of debate. Several studies showed that exosomes isolated from the conditioned medium of cultured cell lines over-expressing Tau or CSF from AD patients indeed contain Tau [26–28], while other studies reported that no Tau was detected in exosomes isolated from conditioned medium of cultured primary neurons or cell lines [12, 29]. Thus, more investigation is needed to clarify this issue.

In the current study, we determined that Tau is a bona fide component of exosomes. We characterized the Tau species secreted in association with exosomes from cultured neurons or human CSF from AD or control subjects. Using microfluidic devices we showed that exosomes play a role in the neuron-to-neuron transmission of Tau. Finally, we found that exosomes could mediate the propagation of Tau aggregation between cells.

## Methods

### Antibodies and chemicals

Mouse monoclonal antibodies against Alix/AIP1 and Flotillin-1 were purchased from BD Biosciences (Heidelberg, Germany). Rabbit polyclonal antibody K9JA was purchased from Dako (Dako, Glostrup, Denmark). Phosphorylation-dependent monoclonal mouse antibody PHF1 (against pS396 + pS404) was a gift from Peter Davies (Albert Einstein College, Bronx, NY, USA); 12E8 (against pS262 and pS356) was from Peter Seubert (Elan Pharmaceuticals, South San Francisco, CA, USA); AT8 (against pS202 + pT205) and AT180 (against pT231) were from Pierce (Thermo, Fisher Scientific, Bonn, Germany). Antibody against GluR1 was purchased from Millipore (Darmstadt, Germany). Thioflavine S and antibody against synaptophysin was obtained from Sigma (Steinheim, Germany).

### Cell culture, transfection and treatments

The inducible Tet-On mouse neuroblastoma cells (N2a) expressing the 4-repeat domain of Tau or full-length Tau harboring the FTDP-17 mutation ΔK280 was generated as previously described [30]. The cells were cultured in Eagle’s Minimum Essential Medium (MEM) supplemented with 10% exosome-depleted fetal bovine serum (FBS), 0.1% nonessential amino acids, and 600 μg/ml G418. The exosome-depleted FBS was prepared by centrifugation at 100,000 × g for 1 h. The expression of Tau was induced with 1 μg/ml doxycycline.

Cortical neurons were isolated from Sprague-Dawley rat embryos at Day 18 (E18) and seeded on poly-D-lysine-coated (50 μg/mL) dishes. The cultures were kept for 4 h in plating medium (MEM, 10% horse serum albumin (no tau was detected in exosomes isolated from 50 ml horse serum, data not shown), 1 mM pyruvic acid, 0.6% glucose, 1× penicillin/streptavidin) and then the medium was exchanged to NeuroBasal medium supplemented with B27 (Invitrogen, Carlsbad, CA, USA), L-Glutamine and Penicillin/Streptomycin. Four days after seeding, cytosine arabinoside (Sigma, Munich, Germany) was added to the conditional medium at a final concentration of 5 μg/ml to inhibit the glial proliferation.

For neuronal culture in microfluidic devices (Xona microfluidics, USA), hippocampal neurons isolated from Sprague-Dawley rat embryos at Day 18 (E18) were seeded at a density of ~6 × 10^4^ cells on one side (somal side). Two weeks later, the other side of the microfluidic devices (neuritic side) was seeded with hippocampal neurons at a density of ~3 × 10^4^ cells. The neurons were cultured as described above.

When the neurons on the somal side of the microfluidic devices reached the age of DIV18 or DIV25, 60 μl of conditioned medium in each well on the somal side was removed and then 10 μl of purified exosomes re-suspended in conditioned medium was added to each of the two wells for 24 h.

Transfections of N2a cells with GFP-Tau construct (human Tau tagged with GFP at the N-terminus (longest isoform in CNS, 2N4R or hTau40, for short Tau^GFP^)) or with GFP-Flotillin (Flotillin^GFP^) and the RFP-Tau construct (human Tau tagged with RFP at the N-terminus (longest isoform in CNS, 2N4R or hTau40, for short Tau^RFP^)) were performed with lipofectamine 2000 (Invitrogen) according to manufacturer’s manual. Twenty-four hours later, the conditioned medium was removed, and the cells were washed with warm PBS and split into new flasks. Cortical neurons were infected with adeno-virus expressing the same Tau construct tagged with CFP at the N-terminus (Tau^CFP^).

### Cytotoxicity assay

Cytotoxicity was determined by a lactate dehydrogenase (LDH) assay kit (Roche Applied Science, Indianapolis, USA) according to manufacturer’s manual. Cell death was calculated as percent of LDH released into the medium, compared with total LDH obtained.

### Organotypic hippocampal slice culture and treatments

Hippocampal organotypic slice cultures were prepared from non-transgenic P8 mice and cultured as described previously [31]. At 15 DIV, 200 μl of a GFP-hTau40-exosome-preparation was applied to the medium of organotypic slice cultures for 24 h at 37 °C/5%CO_2_ atmosphere. As a control, 200 μl of the same exosome preparation was sonicated before application.

### Purification of exosomes

Exosomes were purified from conditioned medium of N2a cells or cultured cortical neurons (DIV14-21) as described [32]. Briefly, conditioned medium was collected from 10 to 16 dishes (150 mm) of neurons at a density of 1.5 × 10^7^ cells/dish or from nine T150 flasks of N2a cells at a density of 2–2.5 × 10^7^ cells/flask. The collected medium was centrifuged at 300 × g for 10 min to remove cells. The supernatant was then sequentially centrifuged at 2000 × g for 10 min to remove dead cells and at 10,000 × g for 30 min to remove cell debris. Afterwards, the supernatant was collected and centrifuged at 100,000 × g for 70 min. The pellet (exosomes + contaminating proteins) was washed with PBS to eliminate contaminated proteins and centrifuged at 100,000 × g for 70 min to collect purified exosomes. The exosomes were then used for treatment of N2a cells or cultured primary neurons.

For sucrose gradient centrifugation, the 100,000 × *g* pellet was resuspended in 2.5 M sucrose in 20 mM Hepes (pH 7.4), and a step gradient of sucrose (2.25, 2.0, 1.75, 1.5, 1.25, 1.0, 0.75, 0.5, and 0.25 M) was layered over the exosome-containing 2.5 M sucrose solution as described [24]. The gradient was spun at 100,000 × *g* for 18 h. Fractions were collected from the bottom of the gradient, diluted with PBS, and spun at 100,000 × *g* for 2 h. The pelleted fractions were then used for immunoblotting.

The sonication of exosomes was done with a Sonoplus Ultrasonic homogenizer (Bandelin, Berlin, Germany) using 20% amplitude for 10s.

### Human CSF collection

Lumbar CSF of patients with AD and from psychiatric controls was collected with an atraumatic needle at the Department of Psychiatry, University Medical Center, Göttingen, Germany after written informed consent was obtained (IRB 02/05/09). All performed analyses with human CSF were approved by the ethics committee of the Medical Faculty, University Medicine Göttingen (IRB 02/05/09). Specimens were collected in polypropylene tubes and centrifuged at 2,000xg for 10 min at room temperature, aliquoted and frozen at −80 °C within 30 min of the procedure’s completion.

All AD patients fulfilled the NINCDS-ADRDA diagnosis criteria for probable AD [33]. Patients were extensively characterized by the memory clinic’s expert physicians and neuropsychologists including physical, neurological, psychiatric and neuropsychological examination, brain imaging as well as CSF Abeta 42, Tau and phospho-Tau analysis. Controls included CSF from patients with different psychiatric diagnoses (overview in Tables 1 and 2). None of the control patients suffered from neurodegenerative disorders or dementia. Only CSF samples with normal routine parameters were used.

**Table 1:** CSF samples from AD patients or control patients with different psychiatric diagnoses used in western blot analysis

Patients	Diagnosis	Gender	Age
502	schizophrenia	m	32
508	alcohol addiction, depression	m	54
511	alcohol addiction, depression	m	49
513	depression	m	60
519	depression	m	47
528	schizophrenia	m	48
534	schizophrenia	f	46
542	schizophrenia	f	63
549	depression	f	44
562	depression	f	55
385	AD	m	82
395	AD	f	77
401	AD	f	66
402	AD	m	75
540	AD	m	78
545	AD	f	77
550	AD	f	69
553	AD	f	81
554	AD	m	74
555	AD	f	87

**Table 2:** CSF samples from AD patients or control patients with different psychiatric diagnoses used in luciferase protein-fragment complementation assay

Patients	Diagnosis	Gender	Age
106	multiple sclerosis	m	57
233	bipolar disorder	f	55
242	seizures	f	70
246	major depression	m	41
253	pseudotumor cerebri	f	46
254	subdural hematoma	m	70
262	alcohol addiction	f	51
270	bipolar disorder	m	59
271	major depression	f	51
273	schizophrenia	f	44
276	ketoacidotic coma, personality change after recurrent ketoacidotic coma	f	53
281	opiate addiction	f	48
308	vascular dementia	f	63
299	AD	f	74
322	AD	f	62
332	AD	m	78
337	AD	m	79
383	AD	m	80
401	AD	f	65
415	AD	f	67
449	AD	m	53
463	AD	m	79
548	AD	m	73
553	AD	f	80
585	AD	f	76
647	AD	f	64

### Purification of extracellular vesicles (EVs) from CSF

EVs were isolated as described previously [23, 34]. In brief, 2 ml of CSF were subjected to subsequent centrifugation steps performed at 4 °C, 3,500 × g for 10 min, 2 times 4500 × g for 10 min, 10,000 × g for 30 min and 100,000 × g for 60 min. The 100,000 × g pellet was washed once with PBS and then re-suspended in PBS.

### Nanoparticle tracking analysis (NTA)

NTA was performed with a NanoSight NS300 (NanoSight Ltd., Amesbury, UK) or a Zetaview instrument (ParticleMetrix Ltd., Meerbusch, Germany). The purified exosomes were diluted 1:100–1:500 (conditioned cell medium) or 1:4 in PBS and subjected to NTA. Samples were measured in triplicates. Particle numbers were analyzed with the Nanoparticle Tracking Analysis (NTA) 3.0 software (for Nanosight NS300) or with the Zetaview 8.02.30.02.

### Flow cytometry

Flow cytometry were performed on a Gallios flow cytometer (Beckman Coulter) equipped with a 22 mW blue laser operating at 488 nm. The enhanced wide forward scatter detector mode (1–19° angles with log amplification) was used to detect the microparticles in conditioned medium. Thirty microliter of conditioned medium containing exosomes was diluted into 1 ml with PBS and then measured on the FITC channel with a flow-rate of 30 μl/min. The instrument was allowed to count to a maximum of 20,000 events or 300 s, whichever was earlier without gating.

### Atomic force microscopy (AFM)

The exosomal samples were diluted in PBS, and then loaded on the freshly cleaved mica for 15 min, followed by rinsing in imaging buffer (10 mm Tris-HCl, pH 7.4, 50 mm KCl). AFM imaging was performed in oscillation mode using a Nano Wizard Ultra-speed AFM microscope (JPK instruments) and Si3N4 cantilevers (NPS series, Bruker) with spring constants of 0.1–0.6 N/m. To achieve minimal imaging forces between AFM stylus and sample, the drive amplitude was set between 0.5 and 1.0 V, and the amplitude set point was adjusted manually to compensate for the thermal drift of the AFM. The acquired images were processed using JPK data processing software. The height analysis was performed using the cross section method in the JPK software.

### Transmission (Cryo-) electron microscopy and tomography

Transmission electron microscopy (TEM) was done as described previously [32]. In brief, exosomes derived from N2a cells or cortical neurons were fixed with 2% PFA and incubated for 20 min on carbon/formvar coated 200 meshes grids. The grids were then washed with PBS for 3 times and treated with 1% glutaraldehyde for 5 min. Afterwards, the grids were embedded in 2% methylcellulose; negative stained with 2% uranyl acetate and analyzed with a Philips CM12 electron microscope (Amsterdam, Netherlands).

For cryo-EM, exosomes were vitrified by plunge freezing (Leica EM GP). In brief, exosomes derived from N2a-cells (expressing Tau^RDΔK280^ or Tau^GFP^) or derived from neuronal cells were loaded onto holey carbon grids (200 mesh, Quantifoil, Germany), followed by plunge-freezing at 95% humidity and 25 °C. The state of vitreous water in the frozen samples was verified by electron diffraction.

**Fig. 1:**
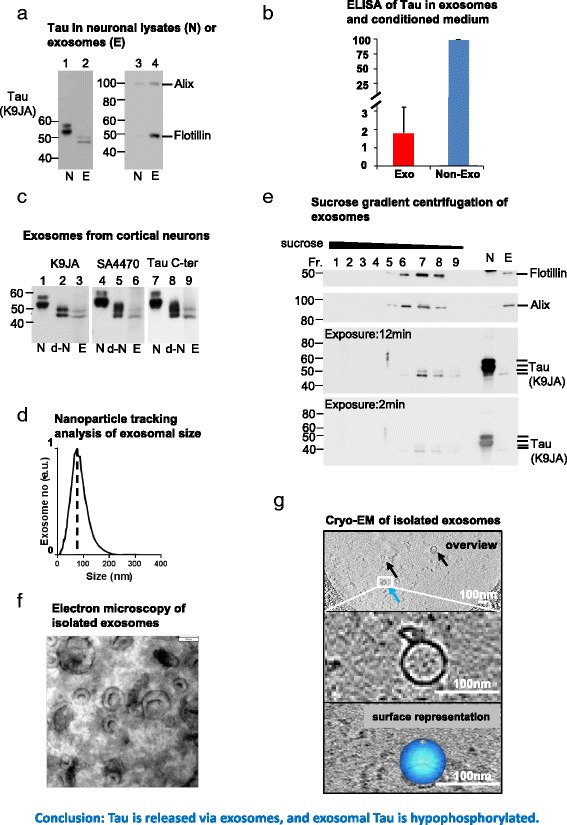
Release of Tau via exosomes by cultured rat cortical neurons. Conditioned medium of cortical neurons (DIV14-21) was collected for isolation of exosomes. N denotes neuronal lysates, E exosomal lysates. **a** Western blot analysis of the distribution of neuronal Tau (*left panel*, lanes 1, 2) and exosomal markers (Flotillin and Alix, right panel, lanes 3, 4) in neuronal lysates and exosomes. Note: (1) Tau typically appears as two bands, a weaker *upper band* at M_r_ ~56kD and a stronger *lower band* at M_r_ ~54kD (lane 1). (2) The Tau bands in exosomes have a *lower* M_r_ (~50kD and 48 kD) than endogenous rat Tau bands. (3) Less Tau was detected in exosomes than in cell lysates, which is in contrast to Flotillin and Alix, indicating Tau is not a major component of exosomes. **b** Quantification of Tau in exosomes and in conditioned medium of neuron culture using ELISA. The conditioned medium from neuron culture (DIV17) was depleted of cells and cell debris via sequential centrifugation at 300 × g, 2000 × g for 10 min and 10,000 × g for 30 min. Tau in conditioned medium represents the total extracellular Tau and was used for calculation of the percentage of exosomal and Non-exosomal Tau. **c** Exosomal Tau is intact and hypophosphorylated: To compare the phosphorylation status of Tau in neuronal lysates and exosomes, neuronal lysates (lane 1) were dephosphorylated with alkaline phosphatase for 30 min at 37 °C (lane 2). This lowers the Mr to the same level as exosomal Tau (lane 3), indicating that the change is due to phosphorylation and not due to truncation. Exosomal Tau is recognized by antibody SA4470 against aa.1-17 of Tau and antibody Tau C-ter against aa.400-441 of Tau (2nd and 3rd panels), indicating that both neuronal and exosomal Tau are intact. **d** Nanoparticle tracking analysis (NTA) of isolated exosomes showing particle number vs. size (arbitrary units, peak = 100%). The distribution peaks at a diameter of ~75 nm, which is typical for exosomes. **e** Isolated exosomes separated by sucrose gradient centrifugation. Neuronal lysates (N) and exosomal lysates (E) were loaded for comparison. Note: (1) Tau is enriched in fractions 7 and 8 coincident with the exosomal markers Flotillin and Alix; (2) the M_r_ values of Tau bands in exosomes are lower than those of endogenous rat Tau bands due to lower phosphorylation, which is consistent with the results shown in A. **f** Negative stain-electron microscopy of isolated exosomes. Many exosomes appear as cup-shaped vesicles with diameters of 40–100 nm. Scale bar = 70 nm. **g** Cryo-electron tomography of isolated exosomes. Because of the better preservation, exosomes reveal a more native spheroidal shape. The *upper panel* (*overview*) shows that most exosomes (*arrows*) exhibit a size of 50–100 nm. For one selected exosome we show here (lower panel) an isosurface representation of the membrane to visualize the outer shape of the vesicle

Cryo-electron tomograms were recorded using a JEOL JEM2200 FS (Figs. 4 and 7) and a FEI Titan Krios (Fig. 1) cryo-Transmission electron microscope at low dose conditions. Tomograms were recorded at magnifications of 14,000x (FEI) and 20,000x (JEOL) corresponding to pixel sizes of 0.5 nm at specimen level, with a defocus of −4 to −6 nm. Samples were tilted from −60 to +60 with tilt increments of 2 or 3° steps, resulting at a total dose of 60–70 e/Å^2^. Tomograms were recorded using serialEM [35, 36]. Tomograms were aligned with respect to a common origin using cross-correlation techniques using the software IMOD [37]. Reconstructions were generated using the Simultaneous Iterative Reconstruction Technique (SIRT) algorithms, and isosurface visualization was performed by the 3dmod software which is part of the IMOD software suite.

### Proteinase K protection assay and high salt treatment of exosomes

The neuron-derived exosomes were divided into 30 μl aliquots and then were either left untreated or treated with 1% saponin, 50 ng proteinase K (prot K) or 50 ng proteinase K together with 1% saponin. The samples were incubated for 5 min or 1 h at 37 °C. SDS loading buffer was then added and the samples were heated at 95 °C for 5 min and analyzed by western blotting.

To dissociate extra-vesicle Tau in association with exosomal surface, the neuron-derived exosomes were incubated with increasing amounts of NaCl (0 M, 0.01 M, 0.1 M, 0.25 M or 0.5 M). Afterwards, exosomes were collected by centrifugation at 100,000 × g, 4 °C for 70 min, then resuspended in SDS loading buffer. The samples were heated to 95 °C for 5 min and analyzed by western blotting.

### Luciferase protein-fragment complementation assay

Complementation pairs of Tau^RDΔK^-N terminus of luciferase and Tau^RDΔK^ –C terminus of Luciferase were transfected into N2a cells in a T25 flask as described above. Three hours after transfection the cells were washed, trypsinated and placed into 96-well plates at a density of 15,000 cells/well. One hour later, the cells were treated with exosomes from human CSF or PBS. Forty-eight hours after treatment, the cells were treated with luciferin for 10 min and then subjected to luciferase activity measurement using an Ivis Lumina II system (Caliper Life Science).

### Immunofluorescence

N2a cells were fixed with 3.7% paraformaldehyde in phosphate buffered saline (PBS) for 15 min at room temperature and then permeabilized with 80% methanol for 6 min at −20 °C. Thioflavine S staining and labeling of Tau was done as described previously [38]. For optical analysis a confocal microscope was used (LSM510, Zeiss, Oberkochen, Germany).

For immunofluorescence of neurons cultured in microfluidic devices, after treatment with exosomes, the chambers were removed and the neurons were fixed with 3.7% Formaldehyde (Sigma)/4% Sucrose in PBS for 30 min at 37 °C. After washing with PBS three times, the cells were permeabilized with 0.1% Triton X-100 for 10 min at room temperature and then blocked in 5% bovine serum albumin (BSA) for 1 h at room temperature, followed by incubation with the primary and secondary antibodies. Confocal images were captured with a LSM700 microscope (Zeiss, Oberkochen, Germany).

Organotypic hippocampal slice cultures were attached on the Millicell membrane and stained as free-floating sections in 6-well plates. Cultures were first fixed with 3.7% paraformaldehyde in PBS for 2 h at 4 °C. After washing with PBS, slices were permeabilized with 0.1% TritonX-100/PBS for 90 min at room temperature. Slices were then blocked with 5% BSA for 2 h, followed by incubation with primary antibodies diluted in PBS for 3 days at 4 °C. After washing three times with PBS, slices were incubated with secondary antibody for 3 days at 4 °C. Afterwards, slices were washed three times with PBS and then mounted with Permafluor mounting solution (Beckman Coulter, Paris, France). The following primary antibodies were used: microtubule associated protein 2a/b (MAP2a/b) (Abcam, 1:1000)), GFAP (Sigma-Aldrich, Germany; (1:1000)) and Iba1 (Wako, Japan 1:500). All fluorescently labeled secondary antibodies were from Dianova (Hamburg, Germany) (1:1000).

### Enzyme-linked immunosorbent assay (ELISA)

The levels of Tau in neuron-derived exosomes and conditioned medium of neuronal culture were determined by a commercially available validated ELISA kit (Invitrogen, Carlsbad, CA, USA) according to the manufacturer’s instructions. All the samples were treated with 1% CHAPS detergent.

### Biochemical analysis

After washing twice with cold PBS, neurons or N2a cells were extracted with extraction buffer (50 mM Tris-HCl, pH 7.5; 100 mM NaCl, 1% Triton X-100 supplemented with phosphatase inhibitors and protease inhibitors). Purified exosomes were extracted with extraction buffer. Afterwards, the extracts were centrifuged at 4 °C at 16,000 × *g* for 20 min, and the supernatants were collected for western blot analysis.

Soluble and insoluble Tau protein was separated by sarkosyl extraction as described previously [38]. For extraction of N2a cells, the sarkosyl-insoluble pellets were suspended in sample buffer in 1/20 of the volume of supernatants, and the sarkosyl insoluble pellets and supernatants were loaded at 60:1 (pellet:supernatant) for western blotting. For extraction of exosomes, the sarkosyl-insoluble pellets were suspended in sample buffer in 1/3 of the volume of supernatants, and the sarkosyl insoluble pellets and supernatants were loaded at 9:1 (pellet:supernatant) for western blotting. Electrophoresis and western blotting were done as described previously [38]. For quantification of Tau levels, the western blots were probed with the pan-Tau antibody K9JA and analyzed by densitometry.

### Statistical analysis

Statistical analysis was performed with the statistics software Prism5 (GraphPad, La Jolla, CA, USA). Data was analyzed either by Student’s t-test or One way ANOVA followed by Tukey’s multiple repeat comparison (Statistica, Statsoft (Europe), Hamburg, Germany) as indicated. Data are shown as mean ± SD or mean ± SEM. *p*-values are as follows:* < 0.05, ** < 0.01, *** < 0.001.

## Results

### The release of Tau via exosomes by cultured cortical neurons

Primary neurons have been shown to release exosomes [39] and recent studies demonstrated the secretion of Tau by different cell lines and neurons [11, 12]. Thus we sought to determine whether Tau protein can be released via exosomes. It was reported that Tau was not contained in exosomes secreted by immature cortical neurons (DIV9) [29]. We confirmed this for exosomes from young neurons (DIV 7-9) (data not shown) and therefore we focused on exosomes released by mature neurons (DIV14-21).

Exosomes in the conditioned medium of the cultured cortical neurons (DIV14-21) were isolated according to a published protocol [32] and lysed for western blot analysis. The isolated exosomes were enriched for Alix and Flotillin--- two well-accepted exosomal markers, as much higher amounts of these two proteins were detected in the exosomal lysates than in the cell lysates (Fig. 1a, lanes 3, 4). Tau was detected in exosomal lysates (Fig. 1a, lane 2), but, unlike Alix and Flotillin, Tau was not a major protein component of exosomes, since its level in exosomal lysates was dramatically lower than that in cell lysates (about 14 fold change, Fig. 1a, lanes 1, 2). The two Tau bands (M_r_ ~ 48 and 50kD) in the exosomal lysates were ~ 5kD smaller than the Tau bands (M_r_ ~ 54 and 56kD) in the cell lysates on SDS gels (Fig. 1a, lanes 1, 2). We suspected that this might be due to the cleavage of Tau at Asp^421^ by caspase 3 because such a truncation generates a Tau fragment ~ 5kD smaller than intact Tau [40]. Thus, exosomal Tau was analyzed with an antibody specific for Tau truncated at Asp^421^, but no signal was detected (data not shown), suggesting that exosomal Tau is not truncated at this site. We quantified Tau in exosomes and in conditioned medium by ELISA. We found that the exosomal Tau accounts for ~2% of the total extracellular Tau (Fig. 1b), consistent with a previous report (~3% of extracellular Tau) [41].

It has been reported that Tau released by cultured neurons is hypophosphorylated [11]. This prompted us to test whether exosomal Tau is also hypophosphorylated and thereby exhibits increased mobility on SDS gels compared to the cytosolic Tau. We examined the phosphorylation status of Tau with phosphorylation-dependent antibody PHF1 and AT8. In addition, we checked if dephosphorylation of neuronal Tau can reduce the Mr difference between neuronal and exosomal Tau. We treated neuronal lysates with alkaline phosphatase (AP) which resulted in a downward Mr shift of ~5kD (Fig. 1c, 1^st^ panel, lanes 1, 2), to a similar level as exosomal Tau bands (Fig. 1c, 1^st^ panel, lanes 2, 3). The loss of phosphorylation was confirmed by the disappearance of the PHF1 and AT8 reactivity (Additional file 1: Figure S1, lanes 1, 2 and 4, 5). Notably, PHF1 and AT8 did not detect Tau bands in exosomal lysates (Additional file 1: Figure S1, lanes 3, 6), indicating that they were not phosphorylated at these sites. Collectively, these results show that exosomal Tau is in a low state of phosphorylation compared to neuronal cytoplasmic Tau.

To further confirm that exosomal Tau is intact but not truncated, we probed Tau with antibody SA4470 (against N-terminal residues 1–17) and with antibody Tau C-ter (against C-terminal residues 400–441) (Fig. 1c, panel 2, 3). Both antibodies recognized exosomal Tau (Fig. 1c, lanes 6, 9), indicating that it is indeed intact.

To characterize the purified neuronal exosomes, we examined the size distribution using nanoparticle tracking analysis (NTA). The size distribution peaked around 75 nm (Fig. 1d), which is typical for exosomes, indicating their enrichment in the preparations. Another characteristic feature of exosomes is their density, ranging from 1.08 to 1.22 g/ml depending on their cellular origin [32]. We fractionated exosomes on a sucrose gradient and examined the distribution of the exosomal markers Flotillin and Alix by western blotting. Both markers were enriched in fractions 5–8 at a density between ~1.16 to ~1.06 g/ml (Fig. 1e, upper panels), which fits well with the density of exosomes. Tau was enriched in fractions 7 and 8 overlapping with Flotillin and Alix (Fig. 1e, bottom panels), suggesting that the majority of Tau is associated with exosomes in these preparations. We assessed the morphology of the purified exosomes using electron microscopy and atomic force microscopy. EM showed that the vesicles were cup-shaped with a diameter of ~40–100 nm (Fig. 1f), consistent with the typical exosomal morphology [32]. AFM confirmed that the majority of the vesicles have diameters around 40–100 nm (Additional file 2: Figure S2). The vesicles exhibited disk-like structures, which is consistent with exosomal morphology reported by other groups [42]. We further analyzed isolated exosomes using cryo-electron tomography, which is known to preserve structures faithfully. This showed that vesicles exhibited a roughly spheroidal shape with diameters of 50 - 100 nm (Fig. 1g), which was in line with exosomal morphology. Collectively, these results demonstrate that Tau can be secreted by neurons via exosomes.

### Upregulation of exosomal Tau release by neuronal activity

**Fig. 2:**
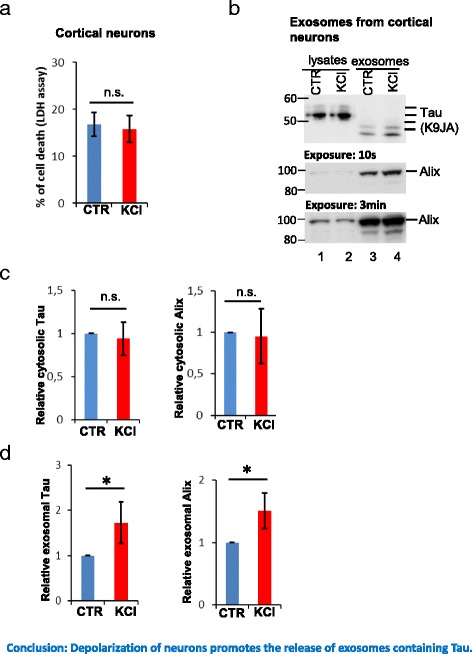
Depolarization of neurons stimulates release of exosomes containing Tau. Cortical neurons were incubated in control medium or depolarizing medium containing 25 mM KCl for 3 h. **a** Cytotoxicity analyzed with LDH release assay. KCl treatment does not cause cell death. Error bars: SD; *n* = 3. Student *t*-test: n.s., not significant. **b** KCl treatment enhances exosomal Tau release. Neurons or exosomes isolated from conditioned medium of control neurons (lanes 1, 3) or depolarized neurons (lanes 2, 4) were lysed for western blot analysis. KCl treatment does not change Tau and Alix levels in neuronal cell lysates (compare lanes 1, 2), but increases both Tau and Alix in exosomes (compare lanes 3, 4). **c**, **d** Quantification of blots shown in B for Tau (upper panel) and the exosome marker Alix (bottom panel) in cell lysates (**c**) or exosomes (**d**). Shown are the ratios of proteins in cell lysates or exosomes (KCl-induced vs. control). Note that upon neuronal activation by KCl, Tau or Alix in exosomes increase by ~50% (**d**), whereas Tau or Alix in cell lysates does not change (**c**). Error bars: SD; *n* = 4. Student *t*-test: *p < 0.05; n.s., not significant

It has recently been reported that the release of endogenous Tau via non-exosomal pathways by primary neurons is a physiological process which is stimulated by neuronal activity [11]. Interestingly, the release of exosomes by neurons is stimulated by neuronal activity as well [39]. Thus, we examined whether stimulating neurons could lead to elevated release of exosomal Tau. Cultured neurons were depolarized with 25 mM KCl for 3 h, while the control cultures were incubated in the same medium containing only 5 mM KCl. We first examined whether depolarization of neurons caused neurotoxicity using the LDH release assay. The high KCl treatment did not increase LDH release (Fig. 2a), suggesting that the depolarization of neurons within the treatment timeframe was not neurotoxic. Next, we checked the amount of Tau and Alix in lysates of these neuronal cultures or exosomes derived from them. In the neuronal lysates, depolarization of neurons did not affect the levels of Tau and Alix (Fig. 2b, lanes 1, 2; Fig. 2c). However, in the exosomal lysates, depolarization of neurons increased both Tau and Alix levels by ~60% (Fig. 2b, lanes 3, 4; Fig. 2d). Since the elevation of Tau was proportional to the elevation of Alix (Fig. 2d), the increase of Tau in exosomal preparations can be taken to result from the increased release of exosomes, instead of selective accumulation of Tau in exosomes. In conclusion, chemical stimulation of neurons increases exosomal secretion, leading to higher release of exosomal Tau.

### Tau is located inside exosomes

**Fig. 3:**
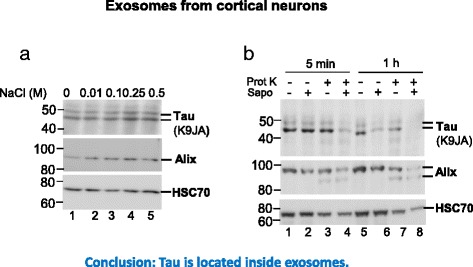
Tau is localized inside exosomes. **a** Neuron-derived exosomes were incubated with increasing concentrations of NaCl to detach proteins peripherally attached to the membrane. Tau is detected with the pan-Tau antibody K9JA. HSC70 and Alix were examined as exosomal markers. Lines on the right indicate Tau protein, Alix and HSC70. M.W. markers are shown on the left. Note that exosomal Tau levels are not changed by NaCl treatment (lanes 1–5), similar to exosomal markers HSC70 and Alix, indicating that Tau is not peripherally attached to the exosomal membrane surface. **b** Proteinase K protection assay. Neuron-derived exosomes treated with or without 50 ng proteinase K (Prot K) in the presence or absence of 1% saponin (Sapo) for 5 min or 1 h at 37 °C, followed by western blot analysis. Note that Tau is strongly reduced (5 min) or even absent (1 h) in exosomes treated with both Prot K and Sapo, compared with treatment with Prot K alone, indicating that the exosomal membrane protects Tau against Prot K digestion

Although a fraction of extracellular Tau is released via exosomes, there is the question of its origin and topology with regard to the exosomal vesicles. For example, since some neuronal death occurs spontaneously during culturing, a concern is whether the exosomal Tau is in fact derived from dead neurons and only loosely attached to the exosomal surface. To clarify this, we treated neuron-derived exosomes with increasing amounts of NaCl (from 0.01 to 0.5 M) to detach Tau stuck on the exosomal outer membrane and checked its influence on Tau and exosomal markers Alix and HSC70 [43]. NaCl treatment had little effect on levels of Alix and HSC70 (Fig. 3a, lower panels), nor on exosomal Tau (Fig. 3a, upper panel). This shows that Tau is not just loosely associated with the exosomal surface, but rather is an integral part of the exosomal vesicle.

To corroborate this finding, we performed a proteinase K protection assay to examine whether Tau is protected by the exosomal membrane. Saponin was used to permeabilize exosomes. The exosomal Tau level was unchanged by treatment with either saponin or proteinase K alone for 5 min (Fig. 3b, upper panel, lanes 1–3), but it was dramatically reduced by ~70% by treatment with proteinase K together with saponin that permeabilized exosomes (Fig. 3b, upper panel, lane 4). The extended treatments with either saponin or proteinase K alone for 1 h reduced exosomal Tau levels by ~50% (Fig. 3b, upper panel, lane 5–7), probably because during incubation, part of the exosomes became broken, leading to the digestion of the released Tau by proteases attached to the exosomal surface or proteinase K resp. Notably, the treatment with saponin and proteinase K for 1 h resulted in the complete digestion of Tau as no exosomal Tau was detected (Fig. 3b, upper panel, lane 8). Collectively, these data show that exosomal Tau is protected from digestion by proteinase K, indicating Tau is located within exosomes. Similarly, the bona fide intraluminal exosomal proteins Alix and HSC70 were protected from proteinase K digestion (Fig. 3b, middle and lower panels, lanes 3, 4, 7, 8).

### Exosomes can mediate the neuron-to-neuron transmission of Tau

**Fig. 4:**
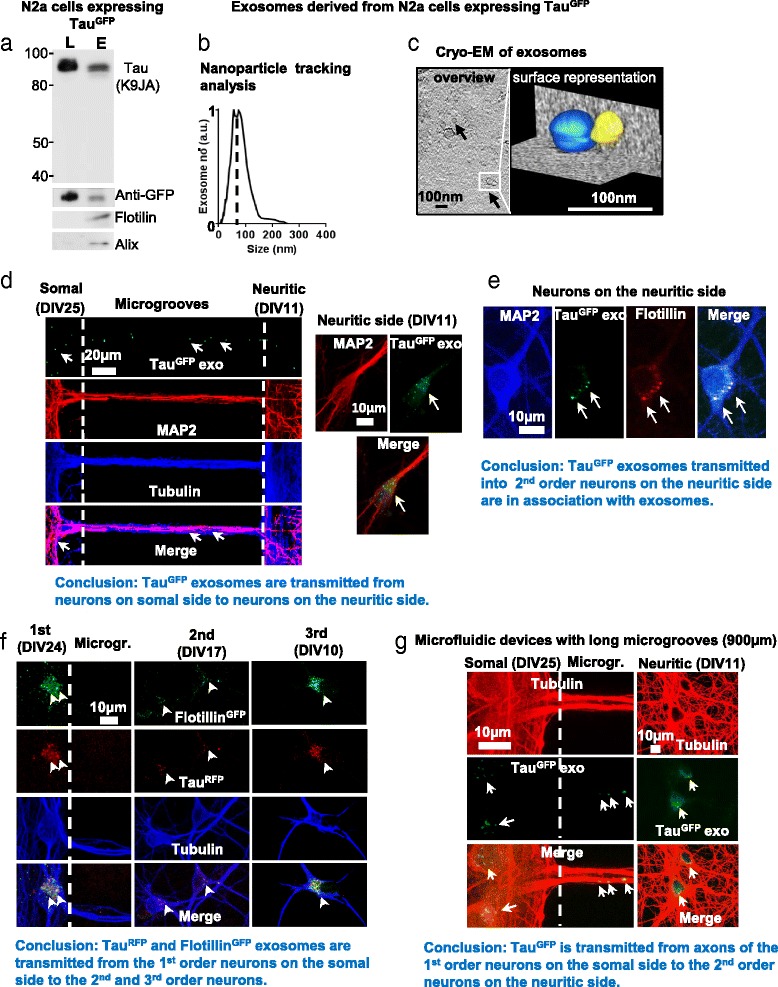
The uptake and trans-synaptic transmission of Tau by cultured rat primary neurons via exosomes in microfluidic chambers. **a** Western blot assay to analyze exosomes isolated from N2a cells transfected with Tau^GFP^ for 2 days. Note that only intact, no truncated Tau^GFP^ fusion protein was detected, and that Tau^GFP^ occurred both in the neuronal and exosomal compartments. **b** Nanoparticle tracking analysis of isolated exosomes. The distribution peaks around ~50 and ~80 nm, indicating the enrichment of exosomes in the preparations. **c** Cryo-electron tomography of isolated exosomes. The left panel (*overview*) shows several exosomes (*arrows*) with an estimated size of ~50–100 nm. The isosurface representation in the *right panel* shows two connected exosomes (the membranes are colored in *blue* and *yellow* respectively). **d** Uptake and transmission of Tau^GFP^ by neurons cultured in microfluidic chambers with short microgrooves (150 μm). The 1^st^ order neurons were treated with Tau^GFP^ exosomes (20 μg) at DIV25 for 24 h, when the 2^nd^ order neurons were at DIV11. Neurons were stained with antibodies against MAP2 (*red*) and tubulin (*blue*). *Arrows* indicate Tau^GFP^ positive vesicles. Note that Tau^GFP^ puncta were detected in the microgrooves (*left panels*) and also in the 2^nd^ order neurons on the neuritic side (*right panels*) that were not treated with Tau^GFP^ exosomes, indicating the uptake and the transmission of Tau^GFP^ via exosomes between the two populations of neurons. Scale bar in *Left panels* = 20 μm; *right panels* = 10 μm. **e** Direct transmission of exosomes from 1^st^ order neurons to the 2^nd^ order neurons in microfluidic chambers. Neurons were treated as described in **d**. Neurons were stained with antibodies against MAP2 (*blue*) and Flotillin (*red*). Scale bar = 10 μm. Arrows indicate Tau^GFP^ exosomes. The colocalization of Tau^GFP^ with Flotillin indicates that Tau is indeed located in the exosomes. **f** Direct transmission of exosomes from 1^st^ order neurons to the 2^nd^ and 3^rd^ order neurons in microfluidic chambers. The 1^st^ order neurons were treated with Tau^RFP^ and Flotillin^GFP^ positive exosomes (20 μg) at DIV24 for 24 h, when the 2^nd^ and 3^rd^ order neurons were at DIV17 and DIV10 respectively. Neurons were stained with antibodies against tubulin (*blue*). Majority of the puncta in 2^nd^ (94.5 ± 3.7% of 220 puncta in 3 different microfluidic chambers) and 3^rd^ populations (85.7 ± 7.1% of 105 puncta in 3 different microfludic chambers) of neurons were positive for both Tau^RFP^ and Flotillin^GFP^. Arrows indicate Tau^RFP^ and Flotillin^GFP^ positive vesicles. Note that Tau^RFP^ and Flotillin^GFP^ positive puncta were detected in the 2^nd^ and 3^rd^ order neurons that were not treated with exosomes, indicating the uptake and the direct transmission of exosomes between the three populations of neurons. Scale bar = 10 μm. **g** The transmission of Tau^GFP^ exosomes from axons of the 1^st^ order neurons to the 2^nd^ neurons cultured in microfluidic chambers with long microgrooves (900 nm). The 1^st^ order neurons were treated with Tau^GFP^ exosomes (20 μg) at DIV25 for 24 h, when the 2^nd^ order neurons were at DIV11. Neurons were stained with an antibody against tubulin (*red*). Scale bar = 10 μm. *Arrows* indicate Tau^GFP^ positive exosomes. Note that Tau^GFP^ puncta were detected in the microgrooves (*left panel*) and also in the 2^nd^ order neurons on the neuritic side (*right panels*) that were not treated with exosomes. Since no dendrites project through the long microgrooves (900 μm) to the neuritic side, the transmission of Tau^GFP^ exosomes occurs through axons of the 1^st^ order neuron to the 2^nd^ order neurons

Since endogenous Tau can be released via exosomes, we next examined whether exosomes can act as carriers to mediate the transmission of Tau between cells. In order to directly monitor Tau-containing exosomes, we prepared exosomes from N2a cells transiently over-expressing human Tau tagged with GFP at the N-terminus (longest isoform in CNS, 2N4R or hTau40, for short Tau^GFP^). Western blot analysis revealed that only intact Tau^GFP^ but not truncated Tau^GFP^ was detected in exosomal preparations (Fig. 4a, top panel), suggesting that all GFP positive exosomes should contain Tau^GFP^. The Tau^GFP^ protein was confirmed by an anti-GFP antibody (Fig. 4a, middle panel). The exosomal preparations were validated by the enrichment of the exosomal marker Flotillin in comparison with the cell lysates (Fig. 4a, bottom panel). Nanoparticle tracking analysis showed that the exosome preparation mainly contained vesicles with a diameter of ~55–80 nm (typical for exosomes), indicating the enrichment of exosomes in the preparations (Fig. 4b). Cryo-electron tomography revealed that the vesicles exhibited a spheroidal shape with a diameter of 50 -100 nm (Fig. 4c), which is in line with exosomal morphology.

To investigate the neuron-to-neuron transmission of exosomal Tau, we took advantage of microfluidic devices (MFD). They allow the culture of two populations of neurons in two separate chambers connected by microgrooves, enabling the direct observation of the transfer of exosomal Tau from neurons on one side (somal side) to neurons on the other side (neuritic side) (Additional file 3: Figure S3, B, D). They also enable the selective treatment of cells on only one side by applying fluidic isolation via maintaining a flow of medium between the two sides (Additional file 3: Figure S3, B, D). One caveat is that the uptake of exosomes can occur both at the somatodendritic and axonal compartments of neurons [6]. Thus even though exosomes were added to the somal side of the chamber, some neurons on the neuritic side projecting through microgrooves might obtain exosomes via direct axonal uptake, independently of the transfer of exosomes from neurons seeded on the somal side. To solve this issue, we seeded the 1^st^ order neurons on the somal side two weeks earlier than the 2^nd^ order neurons on the neuritic side, anticipating that the projections from the 1^st^ order neurons occlude the microgrooves and thus eliminate axons and dendrites from the 2^nd^ order neurons to project through microgrooves (Additional file 3: Figure S3). To test whether indeed only the 1^st^ order neurons project through microgrooves, we selectively stained either the 1^st^ order neurons on the somal side (Additional file 3: Figure S3, C) or the 2^nd^ order neurons on the neuritic side (Additional file 3: Figure S3, A) with DiI and monitored the staining of cells by live imaging. When the 2^nd^ order neurons were stained with DiI, the cell bodies of some of the 1^st^ order neurons on the somal side were also positive for DiI, because their neurites projected through microgrooves into the neuritic side (arrowhead in Additional file 3: Figure S3, A). By contrast, when the 1^st^ order neurons were stained with DiI, no cell bodies of the 2^nd^ order neurons were positive for DiI, although neurites from the 1^st^ order neurons that projected through microgrooves into the neuritic side were labeled by DiI (Additional file 3: Figure S3, C). These results confirm that only the 1^st^ order neurons projected through microgrooves. Accordingly, when the 1^st^ order neurons were treated with Tau^GFP^ exosomes, any Tau^GFP^ found in the neurites or cell bodies of the 2^nd^ order neurons would indicate its transmission from the 1^st^ order neurons.

Treatment of the 1^st^ order neurons on the somal side at DIV25 with Tau^GFP^ exosomes resulted in the distribution of Tau^GFP^ puncta in microgrooves (Fig. 4d, left panels), indicating the uptake of Tau^GFP^ exosomes by the 1^st^ order neurons on the somal side. In addition, in ~4% (4.3 ± 0.4%) of the 2^nd^ order neurons (DIV11), the diffuse distribution of Tau^GFP^ and the accumulation of Tau^GFP^ puncta in cell bodies was observed (Fig. 4d, right panels), suggesting the transmission of Tau^GFP^ from the 1^st^ order neurons to the 2^nd^ order neurons. Notably, the Tau^GFP^ puncta in 2^nd^ order neurons were positive for Flotillin staining (Fig. 4e). Such Tau^GFP^ puncta in 2^nd^ order neurons may come from two sources: (1) Tau^GFP^ exosomes internalized by the 1^st^ order neurons; (2) newly synthesized exosomes in the 1^st^ order neurons which contain Tau^GFP^ released by the internalized exosomes. Thus, we sought to test whether Tau^GFP^ exosomes internalized by the 1^st^ order neurons can be directly transmitted to the 2^nd^ order neurons (option 1). To this end, we isolated exosomes from N2a cells co-transfected with Flotillin^GFP^ and RFP-Tau (Tau^RFP^). We utilized 3-chamber devices, as they allow the observation of the transmission of exosomes to an additional population of neurons in the 3^rd^ chamber. We reasoned that if puncta in the 2^nd^ or 3^rd^ order neurons contain both Tau^RFP^ and Flotillin^GFP^, they would represent the exosomes internalized by the 1^st^ order neurons, as it is unlikely that the newly synthesized exosomes in the 1^st^ or 2^nd^ order neurons encapsulate both Tau^RFP^ and Flotillin^GFP^ released by the internalized exosomes. As shown in Fig. 4f, when the 1^st^ order neurons were treated with Tau^RFP^ and Flotillin^GFP^ exosomes, majority of puncta in the 2^nd^ order neurons were positive for both Tau^RFP^ and Flotillin^GFP^. In addition, puncta containing Tau^RFP^ and Flotillin^GFP^ were also observed in the 3^rd^ order neurons. These results suggest that exosomes can indeed be directly transmitted between neurons.

Since recent studies have suggested that the spreading of Tau occurs by a trans-synaptic mechanism [8, 9, 44], we sought to determine whether and how the transmission of Tau^GFP^ exosomes can occur from the axons of the 1^st^ order neurons to the 2^nd^ order neurons. The microfluidic devices with short microgrooves (~150 μm) used in the above experiments allow axons and dendrites of the 1^st^ order neurons to reach the neuritic side, enabling the potential transfer of Tau^GFP^ exosomes to 2^nd^ order neurons. To distinguish between axons and dendrites, we performed experiments in microfluidic devices with long microgrooves (~900 μm) that prevent dendrites to project to the neuritic side. Tau^GFP^ puncta were detected in both microgrooves and 2^nd^ order neurons (Fig. 4g), indicating that transmission of Tau^GFP^ can occur through axons.

### Synaptic contacts are required for exosome-mediated transmission of Tau

**Fig. 5:**
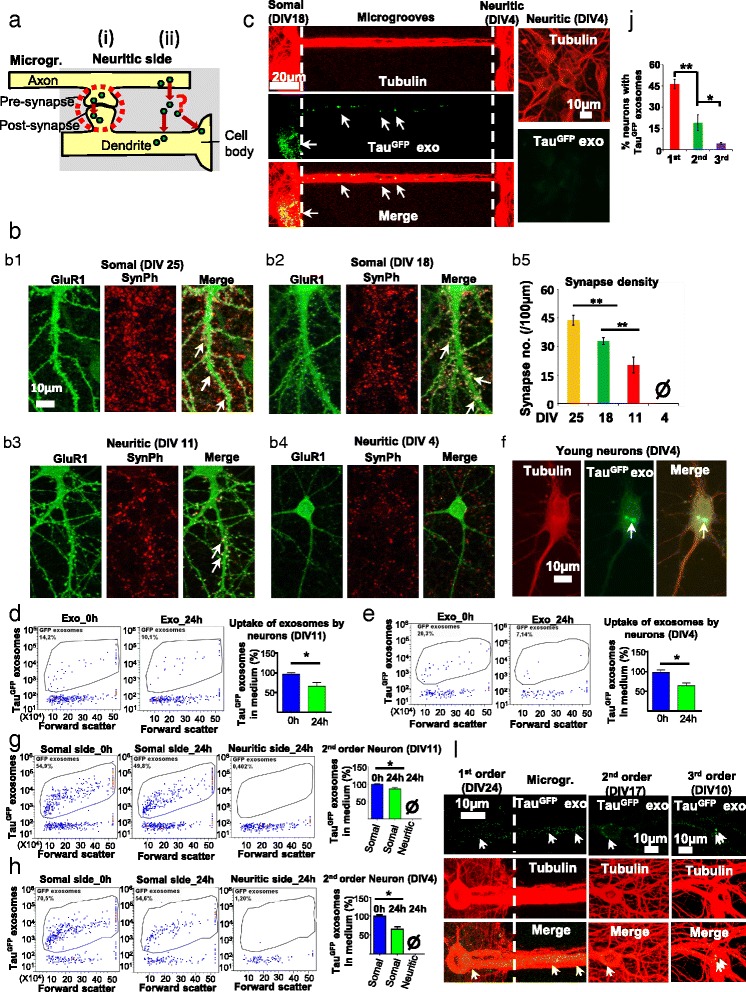
Synaptic contacts are required for exosome-mediated transmission of Tau^GFP^. **a** Diagram illustrating the possible mechanisms underlying exosome-mediated Tau transmission. (i) Transmission occurs specifically through trans-synaptic connections from 1^st^ order to 2^nd^ order neurons. (ii) Exosomes in 1^st^ order neurons are released into the conditioned medium and then internalized by 2^nd^ order neurons. **b** Formation of synaptic contacts at different states of maturation. (b1, b2) Neurons were stained for post-synaptic marker GluR1 (*green*) and pre-synaptic marker synaptophysin (SynPh) (*red*). The co-localization of GluR1 and synaptophysin indicates formation of synaptic contacts (*white arrows* on *right panel*). Synaptic connections were formed in the 1^st^ order mature neurons (DIV 25 or DIV18) on the somal side (b1, b2) and also in the 2^nd^ order old neurons on the neuritic side (DIV11) (b3), but not in the 2^nd^ order young neurons (DIV4) (b4). Scale bar = 10 μm. (b5) Quantification of synapse density shown in b1-b4. Synapses are formed in neurons at DIV11, although the density is lower than that in mature neurons (DIV18 and 25). Notably, nearly no synapses are formed in young neurons at DIV4. Error bars: SEM; *n* = 3 in b5. Student *t*-test: **p < 0.01. **c** No transmission of Tau^GFP^ exosomes from mature neurons (DIV18) to very young neurons (DIV4) cultured in microfluidic chambers. The 1^st^ order neurons were treated with Tau^GFP^ exosomes (20 μg) at DIV18 for 24 h, when the 2^nd^ order neurons were at DIV4. Neurons were stained with an antibody against tubulin (*red*). Scale bar in left panel = 20 μm; in right panel = 10 μm. Arrows indicate Tau^GFP^ positive exosomes in the microgrooves (*left side*, *middle*), but not in the 2^nd^ order neurons on the neuritic side (*right side*, *bottom*) that were not treated with exosomes, indicating no transmission of Tau^GFP^ via exosomes between the two populations of neurons. **d**, **e** FACS analysis of the uptake of Tau^GFP^ exosomes by young (DIV4) and old neurons (DIV11). Neurons were treated with Tau^GFP^ exosomes (20 μg) for 24 h. Conditioned medium was collected immediately after the addition of Tau^GFP^ exosomes (Exo-0 h) or 24 h later (Exo-24 h) for flow cytometry. The *left* and *middle panels* show a representative readout of the flow cytometry. *Right panel*: quantification of 3 experiments. Note that Tau^GFP^ exosomes in conditioned medium is dramatically reduced after 24 h treatment, indicating the uptake of these exosomes by young and old neurons (DIV4 or DIV11). Error bars: SD; *n* = 4 and *n* = 3 in **d** and **e** respectively. Student t-test: *p < 0.05. **f** The internalization of Tau^GFP^ exosomes by young neurons (DIV4). Young neurons (DIV4) were treated with Tau^GFP^ exosomes (20 μg) for 24 h and then stained with an antibody against tubulin (*red*). Scale bar = 10 μm. Arrows indicate Tau^GFP^ positive exosomes. Note that Tau^GFP^ puncta are detected inside neurons, suggesting the uptake of Tau^GFP^ exosomes by young neurons. **g**, **h** FACS analysis showing that there is no release of Tau^GFP^ exosomes into conditioned medium on the neuritic side. The 1^st^ order neurons cultured on the somal side in microfluidic chambers were treated with Tau^GFP^ exosomes (20 μg) for 24 h. Conditioned medium on the somal side or neuritic side was collected immediately after the addition of Tau^GFP^ exosomes (Exo-0 h) or 24 h later (Exo-24 h) for flow cytometry. The *left 3 panels* show a representative readout. The *right panels*: quantification of 3 experiments. Almost no Tau^GFP^ positive exosomes were detected in the conditioned medium on the neuritic side, indicating that the internalized Tau^GFP^ exosomes were not released into the conditioned medium on the neuritic side by the 1^st^ order neurons. Error bars: SD, *n* = 3. Student *t*-test: ** *p* < 0.01. **i** Transmission of Tau^GFP^ exosomes from 1^st^ order neurons to the 2^nd^ and 3^rd^ order neurons in triple-chamber devices. The 1^st^ order neurons cultured in the 1^st^ chamber were treated with Tau^GFP^ exosomes (20 μg) at DIV24 for 24 h. Neurons were stained with an antibody against tubulin (*red*). *Arrows* indicate Tau^GFP^ exosomes. Note the presence of Tau^GFP^ exosomes in all the three population of neurons indicating transmission of Tau^GFP^ via exosomes between the three different populations of neurons. Scale bars = 10 μm. **j** Quantification of the percentage of neurons in all the three channels with Tau^GFP^ exosomes. Error bars: SEM; *n* = 3. Student *t*-test: * *p < 0.05,* **p < 0.01

We next determined how the Tau^GFP^ exosomes were transmitted from 1^st^ order neurons to 2^nd^ order neurons in microfluidic devices. Two possible mechanisms may explain the exosome-mediated transmission of Tau. (i) Transmission takes place specifically through trans-synaptic connections from 1^st^ order to 2^nd^ order neurons. (ii) Exosomes in 1^st^ order neurons are released into the conditioned medium and then internalized by 2^nd^ order neurons at extrasynaptic sites (Fig. 5a). We analyzed the formation of synapses in 2^nd^ order neurons (DIV11) on the neuritic side by examining the co-localization of the post-synaptic marker GluR1 (green) and the pre-synaptic marker synaptophysin (red) (Fig. 5b). Mature synapses were observed in 2^nd^ order neurons (DIV11) (Fig. 5, b3), although at a lower density than in mature neurons (DIV 25 or DIV18) on the somal side (Fig. 5, b1, b2, b5). Thus, synaptic contacts may contribute to Tau^GFP^ transmission in this case. To further determine the potential role of synaptic contacts in the transmission of Tau, we examined whether the transmission of Tau^GFP^ exosomes can occur when no synapses are present in 2^nd^ order neurons. To this end, we selectively treated the 1^st^ order neurons (DIV18) when the 2^nd^ order neurons were at a very early stage (at DIV4). No synaptic contacts were detected in 2^nd^ order neurons at DIV4 (Fig. 5, b4). Notably, no Tau^GFP^ puncta were detected in the cell bodies of the 2^nd^ order neurons at DIV4 (before synapse formation, Fig. 5c, right panels), although Tau^GFP^ puncta in microgrooves were detected (Fig. 5c, left panels). This indicates that although Tau^GFP^ exosomes were taken up by the 1^st^ order neurons on the somal side, they were not transmitted to 2^nd^ order neurons. Taken together, these results suggest that synaptic contacts are necessary for the transmission of Tau by exosomes.

The low transmission of Tau into young neurons (DIV4) could be explained either because these neurons have not yet developed an uptake mechanism for exosomes, or because the Tau^GFP^ exosomes were not released on the neuritic side by the 1^st^ order neurons. To clarify these alternatives we examined the uptake of exosomes by the young neurons (DIV4) and the release of the Tau^GFP^ exosomes on the neuritic side by neurites of 1^st^ order neurons projecting through microgrooves using flow cytometry. To analyze the uptake of exosomes, young neurons (DIV4) or old neurons (DIV11) cultured in 24 well-plates were treated with purified Tau^GFP^ exosomes and the conditioned medium was collected for flow cytometry immediately after the addition of exosomes (Exo_0h) or 24 h later (Exo_24 h). After 24 h treatment, the amount of Tau^GFP^ exosomes in the conditioned medium was strongly reduced for both young neurons (DIV4) (down to 64.9 ± 12.7% of Exo_0h, Fig. 5e) and old neurons (DIV11) (to 57.3 ± 9.8% of Exo_0h, Fig. 5d), indicating that the uptake of Tau^GFP^ exosomes occurs at similar rate in both young (DIV4) and old (DIV11) neurons.

We further checked the uptake of Tau^GFP^ exosomes in young neurons (DIV4) by immunofluorescence assay. Tau^GFP^ exosomes were detected in neurons (arrows in Fig. 5f), indicating the internalization of exosomes. To determine the release of Tau^GFP^ exosomes by the neurites of the 1^st^ order neurons on the neuritic side, conditioned medium on the somal and neuritic sides of the microfluidic chamber were collected for flow cytometry immediately after (Exo_0h) or 24 h after the addition of exosomes at the somal side (Exo_24 h). Nearly no Tau^GFP^ exosomes were detected in conditioned medium on the neuritic side after 24 h treatment of 1^st^ order neurons on the somal side with Tau^GFP^ exosomes (Fig. 5g, h), regardless of the age of the 2^nd^ order neurons (DIV4 or DIV11). This result suggests that once Tau^GFP^ exosomes were internalized by the 1^st^ order neurons, they were rarely released into conditioned medium on the neuritic side by neurites of the 1^st^ order neurons. Thus the transmission of Tau^GFP^ from the 1^st^ order neurons to the 2^nd^ order neurons (DIV11) occurs through synaptic contacts: Tau^GFP^ exosomes are released by presynapses and taken up by postsynapses.

To highlight the importance of synaptic connections for the transmission of Tau by exosomes, we performed another set of experiments using 3-chamber devices. When neurons were plated in all the three chambers, treatment of the neurons in the 1^st^ chamber with Tau^GFP^ exosomes resulted in the distribution of Tau^GFP^ puncta in ~20% (20% ± 12.9%) of 2^nd^ order neurons and ~4% (4.5 ± 0.5%) of 3^rd^ order neurons. This suggests that Tau^GFP^ was transmitted (Fig. 5 i, j). However, when neurons were plated in the 1^st^ and 3^rd^ chambers, but not 2^nd^ chambers (Additional file 4: Figure S4, A) (so that the axons from the two populations of neurons projected to the 2^nd^ chamber), no synapses were formed between them. Treatment of the neurons in the 1^st^ chamber with Tau^GFP^ exosomes resulted in the distribution of Tau^GFP^ puncta in microgrooves between 1^st^ chamber and 2^nd^ chamber (Additional file 4: Figure S4, B), indicating the uptake of Tau^GFP^ exosomes by neurons in the 1^st^ chamber. However, no Tau^GFP^ exosomes were detected in microgrooves between the 2^nd^ and 3^rd^ chamber and in neurons in the 3^rd^ chamber. This means that no transmission of Tau^GFP^ occurred between the two populations of neurons (Additional file 4: Figure S4, B). The results confirm that synaptic contacts are necessary for the transmission of Tau by exosomes.

Collectively, these data argue that exosomes can mediate the trans-synaptic transmission of Tau. Notably, when the 1^st^ order neurons were treated with exosomes that were disrupted by sonication, no Tau^GFP^ puncta were detected in the microgrooves and the 2^nd^ order neurons (data not shown), indicating that the integrity of exosomes is necessary for the transmission of Tau protein.

To exclude the possibility that the transmission of exosomal Tau between neurons is only applied to exosomes derived from N2a cells, we treated neurons in microfluidic devices with exosomes derived from neurons infected with adeno-virus expressing CFP-Tau40 (Tau^CFP^). Similar to treatment with N2a cell-derived exosomes, CFP puncta were observed in the 2^nd^ order neurons (Additional file 5: Figure S5B, right panels), suggesting exosomes can indeed serve as a carrier to regulate the transfer of Tau between neurons independently of the origin of exosomes.

**Fig. 6:**
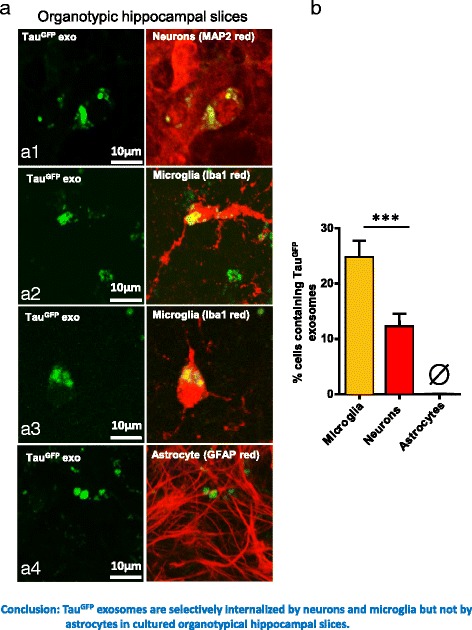
Uptake of Tau^GFP^ exosomes by neurons and microglia in cultured organotypic hippocampal slices. **a** Immunofluorescence assay showing the uptake of Tau^GFP^ exosomes by hippocampal slice cultures. Hippocampal slice cultures were incubated for 24 h with exosomes (20 μg) isolated from N2a cells expressing Tau^GFP^. Cultures were afterwards stained for neuronal marker MAP2 (*red*, a1), microglial marker Iba1 (*red*, a2, a3) or astrocytic marker GFAP (*red*, a4). Tau^GFP^ colocalizes with MAP2 (A1) and Iba1 (a2, a3) suggesting the uptake of exosomes by neurons (a1) and microglia (a2, a3). Nearly no colocalization of Tau^GFP^ with GFAP is observed (a4), indicating no internalization of exosomes by astrocytes. Scale bars = 10 μm. **b** Quantification of the uptake of Tau^GFP^ exosomes by neurons, microglia and astrocytes shown in (**a**). Significantly higher percentage of microglia (24.8 ± 14.1%) than neurons (12.3 ± 11.2%) exhibits uptake of exosomes (*n* = 22–24 slices). Nearly no uptake of exosomes is observed in astrocytes. Error bars: SEM. ANOVA: *** *p* < 0.001

We also tested the uptake of Tau^GFP^ exosomes in organotypic hippocampal slice cultures. As shown in Fig. 6a, we observed co-localization of Tau^GFP^ with the neuronal marker MAP2 (a1) and microglial marker Iba1 (a2, a3) but not with the astrocytic marker GFAP (a4), indicating Tau^GFP^ exosomes were preferentially internalized by neurons and microglia. Microglia are more competent in uptake of exosomes than neurons, as higher percentage of microglia (24.8 ± 14.1%) than neurons contained Tau^GFP^ exosomes (Fig. 6b). When the exosomes were disrupted by sonication, no uptake of Tau^GFP^ was observed (Additional file 6: Figure S6), consistent with the observation in primary neurons, indicating that exosomes need to be intact for internalization to occur.

### The release of aggregation-prone Tau via exosomes by an N2a cell model of Tau aggregation

Several recent studies showed that pre-formed Tau aggregates can be taken up by cultured cells and subsequently induce local accumulation of other Tau molecules [14, 15]. We asked whether Tau aggregates can be encapsulated into exosomes and subsequently mediate the propagation of Tau aggregation. To this end, we made use of a cell model overexpressing the pro-aggregant 4-repeat domain of Tau with the mutation ΔK280 (Tau^RDΔK^), since Tau forms aggregates in this cell model [30, 38].

**Fig. 7:**
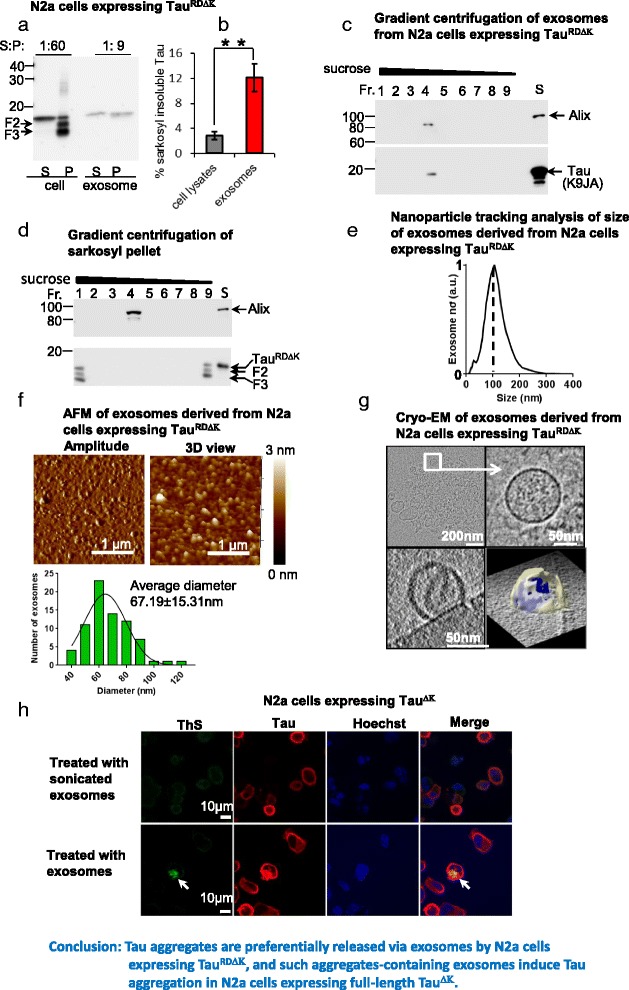
Tau aggregates are preferentially released via exosomes by a cell model of tauopathy, which can induce aggregation of Tau in N2a cells. Tet-inducible N2a cells were induced to express Tau^RDΔK^ for 2 days by addition of doxycyclin. Then the cells were harvested for sarkosyl extraction to separate soluble Tau and insoluble Tau. The conditioned medium was collected for isolation of exosomes. S and P denote supernatant and pellet of sarkosyl extraction respectively. **a** Tau^RDΔK^ aggregates in N2a cells and in exosomes. Soluble Tau and Tau aggregates in N2a cells expressing Tau^RDΔK^ or exosomes from this cell model were separated by sarkosyl extraction. The protein loading ratio between supernatant and pellet for N2a cells was 1:60, for exosomes was 1:9. Note that fragment F2 and F3 were detected in pellet of cell extracts but not in pellet of exosomal extracts. **b** Quantification of Tau^RDΔK^ shown in (**a**). Note that much more Tau^RDΔK^ aggregates were detected in exosomes than in N2a cells. Error bars: SD; *n* = 3. Student t-test: ** *p* < 0.01. **c** Analysis of isolated exosomes separated by sucrose gradient centrifugation. Tau^RDΔK^ appears in fraction 4, coincident with the exosomal marker Alix. **d** Analysis of sarkosyl pellet of cell lysates separated by sucrose gradient centrifugation. Tau^RDΔK^ is enriched in fraction 1 and 9, but not in fraction 4-- the fraction shown to contain exosomes in (**c**). This indicates that the presence of Tau^RDΔK^ in exosomes is not due to the contamination of Tau aggregates. **e** Nanotracking analysis of isolated exosomes. The size distribution peaks at ~100 nm, which is typical for exosomes. **f** Atomic force microscopy of isolated exosomes. The diameters of the majority of the vesicles are 40–100 nm. The average diameter of the exosomes isolated from N2a cells expressing Tau^RDΔK^ is 67.2 ± 15.3 nm and the average height is 2.2 ± 0.8 nm. The exosomes appear to be disk-like shaped. **g** Cryo-electron tomography of isolated exosomes (compare Fig. 1g). **h** Induction of aggregation of full-length Tau^ΔK^ in N2a cells by exosomes from N2a cells expressing Tau^RDΔK^. N2a cells were transfected with pro-aggregant Tau^ΔK^ and induced to express the protein for 1 day. Then the cells were treated with exosomes (20 μg) derived from N2a cells expressing Tau^RDΔK^ and continuously induced to express Tau^ΔK^ for additional 2 days. Tau^ΔK^ was labeled with K9JA antibody (*red*). Thioflavine S staining was performed to monitor Tau aggregates (*green, arrow*). Hoechst staining was used to label nuclei (*blue*). Note that ThS positive cells were detected in N2a cells (~20-30/3-4X10^4^ cells, repeated 3 times) treated with exosomes containing Tau^RDΔK^, but not in N2a cells treated with broken exosomes. *Arrow* indicates Tau aggregates. Scale bars = 10 μm

We isolated exosomes from conditioned medium of this cell model and separated the soluble Tau and Tau aggregates using sarkosyl extraction. The sarkosyl extracts were used as controls. Consistent with our previous studies [38, 45], the sarkosyl-insoluble pellet from the cell lysates contained not only the protein Tau^RDΔK^ but also two smaller fragments (F2 and F3) which are generated by endoproteases and are required for nucleated aggregation (Fig. 7a). By contrast, the exosomal lysates contained only Tau^RDΔK^, but fragments F2 or F3 are below detectability (Fig. 7a). In cell lysates, only ~3% of Tau was insoluble, while in exosomal extracts insoluble Tau was increased to ~12% (Fig. 7b). This result indicates that insoluble Tau is enriched in exosomes compared to cell lysates.

Since high speed centrifugation was used to isolate exosomes from the conditioned medium of N2a cells, this raises the question whether Tau aggregates detected in exosomal preparations actually represent the centrifugation-precipitated Tau aggregates derived from dead cells. To clarify this issue, we performed sucrose gradient centrifugation to fractionate the exosomal preparations and the sarkosyl insoluble pellet of the cell lysates, resp. and then examined whether Tau aggregates exhibit the same density as exosomes. For exosomal preparations, both exosomal marker Alix and Tau were only detected in fraction 4 with a density of ~1.19 g/ml (Fig. 7c). This is consistent with exosomal density (1.08 to 1.22 g/ml), indicating our preparations were enriched in exosomes. For the sarkosyl insoluble pellet of cell lysates, Tau was mainly found in fractions 1 and 9 (Fig. 7d, lower panel, lane 1, 9). The Tau in fraction 9 may be the soluble species that resulted from Tau aggregates, owing to the equilibrium between soluble and insoluble Tau. No Tau was detected in the exosomes-enriched fraction 4 (Fig. 7d, lower panel, lane 4). Of note, Alix was detected in fraction 4 (Fig. 7d, upper panel, lane 4), which might derive from intracellular vesicles contaminating the pellet of sarkosyl extraction. Collectively, these data indicate that the presence of Tau^RDΔK^ in exosomes is not due to the contamination of Tau aggregates in the exosomal preparation.

We further characterized isolated exosomes from N2a cells with nanoparticle tracking analysis, which showed that they mainly contained vesicles with a diameter of around 100 nm (typical for exosomes) (Fig. 7e). This was confirmed by AFM, which showed that the size of most of the vesicles was 40–100 nm (Fig. 7f). Finally, the isolated exosomes were analyzed with cryo-EM. The vesicles exhibited a spheroidal shape with a diameter of ~100 nm (Fig. 7g), which was consistent with exosomal morphology. Interestingly, materials of high density (indicated in blue, Fig. 7g, right lower panel) were detected in the vesicles. However, whether they represent Tau aggregates is not certain.

### Exosomes derived from N2a cells expressing aggregation-prone Tau^RDΔK^ induce aggregation of full-length Tau in N2a cells

Given that exosomes derived from N2a cells expressing Tau^RDΔK^ contain insoluble Tau, we asked whether such exosomes could induce the aggregation of full-length Tau molecules in N2a cells. We previously reported that overexpressing aggregation-prone Tau fragments (e.g. fragment F3 = Tau_258–360_) can induce the aggregation of full-length Tau with the ΔK280 mutation (Tau^ΔK280^) [38, 45]. By analogy, we tested whether exosomes derived from N2a cells expressing Tau^RDΔK^ can induce the aggregation of hTau^ΔK^. We monitored Tau aggregates using thioflavine S staining. The treatment by such exosomes indeed induced aggregation in N2a cells expressing Tau^ΔK^ (~20-30/3-4X10^4^ cells) (Fig. 7h, lower panel). Of note, treatment with sonicated exosomes failed to induce Tau aggregation (Fig. 7h, upper panel). These results demonstrate that exosomes can mediate the propagation of Tau aggregation.

### Exosomes in CSF from AD patients and control subjects contain Tau and can induce Tau aggregation in cultured N2a cells

Given that Tau can be released by primary neurons via exosomes, we further searched for in vivo evidence of exosomal Tau. To this end, we isolated exosomes from the CSF of AD patients or control subjects (Table 1) and examined whether Tau was a component of such exosomes. The purification of exosomes by this method was validated with sucrose gradient centrifugation, revealing the presence of the exosomal marker protein Flotillin at a density of 1.16–1.24 g/ml [23] and electron microscopy [34]. We first characterized the isolated exosomes using nanoparticle tracking. The size distributions of the particles showed maxima around 70–100 nm (Additional file 7: Figure S7), indicating that the preparations were indeed enriched in exosomes.

**Fig. 8:**
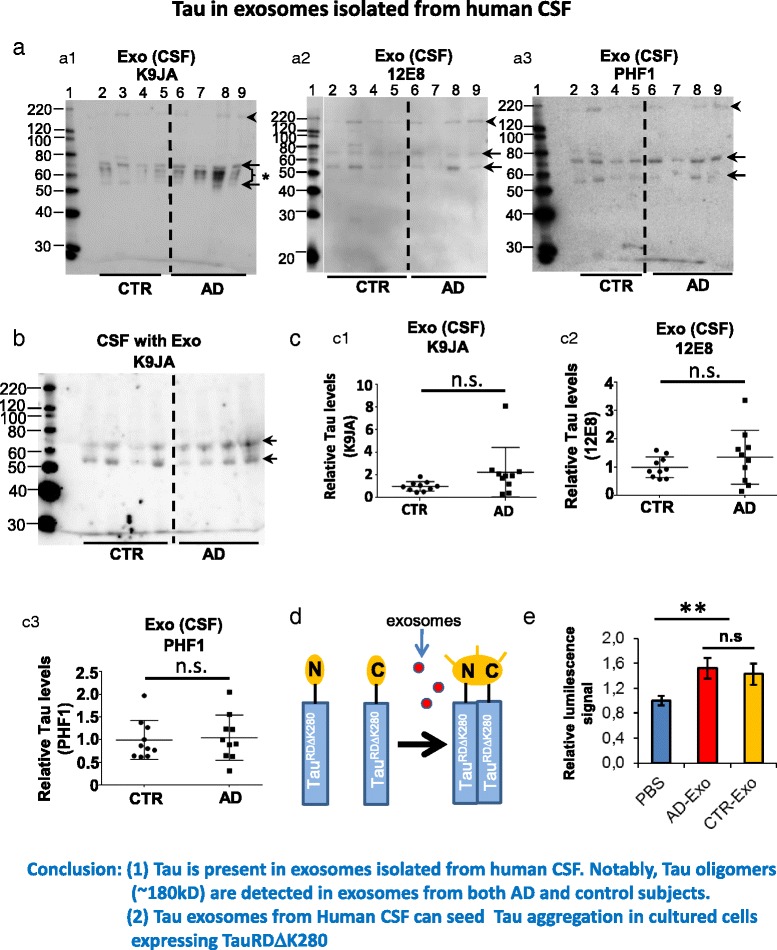
Exosomes isolated from CSF of AD or control subjects contain Tau and induce Tau^RDΔK^ aggregation. **a** The presence of Tau monomers and trimers in exosomes determined with western blots. Besides Tau monomers, Tau trimers (indicated by *arrowheads*, *top right*) ran around 180kD were detected in both exosomes from AD and controls. These Tau trimers were phosphorylated at 12E8 sites (a2) and PHF1 (a3). Two Tau monomer bands were also phosphorylated at PHF1 sites and 12E8 sites (indicated with *arrows*). Between the phosphorylated Tau monomer bands, other Tau bands were not phosphorylated at PHF1 and 12E8 sites (a1, indicated by *). **b** Tau in CSF before isolation of exosomes. Monomeric Tau (doublet between Mr 55-70kD (indicated by *arrows*), but no Tau oligomers of ~180kD was detected. **c** Quantification of Tau shown in **a**. No significant differences of the amount of total Tau (c1) and Tau phosphorylated at 12E8 sites (c2) and PHF1 sites (c3) were observed between exosomes isolated from AD and controls. Error bars: SD. Student *t*-test: n.s., not significant. **d** Diagram showing the luciferase protein-fragment complementation assay. N2a cells were co-transfected with Tau^RDΔK^ fused to split luciferase constructs (N: N-terminal part of luciferase, C: C-terminal part of luciferase). A luminescence signal is only detected after complementation of both split luciferase fragments (e.g. during dimerization and oligomerization of Tau^RDΔK^) and indicates the induction of Tau^RDΔK^ aggregation. **e** Promotion of Tau^RDΔK^ aggregation by exosomes from human CSF. Luminescence increases in reporter cells upon treatment with exosomes prepared from equal volumes of CSF from AD (*n* = 13) or controls (*n* = 13). All measurements were performed in triplicates. **, *p <* 0.001, n.s., non-significant, one way ANOVA

Western blot analysis with pan-Tau antibody K9JA revealed multiple bands between 53kD and 80kD in both AD and control exosomal preparations (Fig. 8, a1). A further band of ~180kD (arrowhead in Fig. 8, a1-a3) was detected in both AD and control exosomal preparations, indicating the presence of SDS-stable Tau oligomers in exosomes. To determine whether the Tau oligomers were selectively encapsulated into exosomes, we checked Tau in CSF before exosome isolation. Two Tau bands above 50kD and 60kD were detected, but no band around 180kD (Fig. 8b), suggesting that the Tau oligomers were enriched in exosomes, compared to the free CSF Tau. No significant differences in the levels of Tau was observed between exosomes from AD and from controls (Fig. 8, c1). Further analysis of Tau phosphorylation revealed that the ~180kD band (arrowhead in Fig. 8, c2, c3) and two other Tau bands (~53kD and ~68kD, arrows) were phosphorylated at the 12E8 and PHF1 sites (Fig. 8, c2, c3). This is in contrast to Tau in exosomes from cultured neurons which is hypophosphorylated based on the increased mobility during gel electrophoreses (Fig. 1c). No significant differences in the levels of Tau phosphorylated at 12E8 and PHF1 sites was detected between exosomes from AD and from controls (Fig. 8, c2, c3). Notably, multiple Tau bands between 53kD and 68kD (Fig. 8, a1, indicated by *) were not recognized by 12E8 and PHF1 antibodies (Fig. 8, a2, a3).

Taken together, these data suggest that both monomeric and oligomeric Tau phosphorylated at 12E8 and PHF1 sites as well as monomeric Tau unphosphorylated at 12E8 and PHF1 sites can be secreted via exosomes in vivo in human brains, and in both cases the Tau monomers or oligomers are encapsulated within the exosomes.

Recently, it was shown that exosomes from patients with Parkinson Disease (PD) and Dementia with Lewy Bodies (DLB) can induce α-synuclein aggregation [34]. This prompted us to examine whether exosomes isolated from human CSF are able to seed Tau aggregation, since we showed above that these exosomes contain Tau oligomers (Fig. 8a), and that exosomes isolated from N2a cells expressing Tau^RDΔK^ can induce Tau aggregation (Fig. 7h). We took advantage of a split-luciferase fragment complementation technique to analyze whether exosomes from human CSF can promote Tau^RDΔK^ aggregation (Fig. 8d). Treatment with exosomes from AD or control CSF (Table 2) dramatically increased Tau^RDΔK^ aggregation, as they elevated the luminescence signal by ~50 and 40% respectively compared to control treated with PBS (Fig. 8e). Slightly higher aggregation was induced by exosomes from AD than exosomes from controls, but this did not reach statistical significance (Fig. 8e).

## Discussion

Tau pathology spreads in a hierarchical pattern in AD. It has been proposed that this can occur through the direct transfer of Tau between neurons through neuronal synaptic connections [8, 9], but the mechanism underlying this process remains elusive. Here, we demonstrate that exosomes can act as carriers to mediate the direct trans-synaptic transmission of Tau.

In cultured primary neurons, the secretion of Tau has been proposed to partly depend on pre-synaptic vesicle secretion [11], however, Tau is not found in the synaptic vesicle proteome [46]. Other mechanisms thus must be involved in the secretion of Tau. Growing evidence suggests that Tau is released via an unconventional secretory pathway independent of the ER/Golgi mediated secretory pathway [12]. This pathway includes 4 modes: direct translocation from the cytoplasm across the plasma membrane, release via secretory lysosomes, microvesicle shedding, or exosome release [47]. It has been a matter of debate whether Tau is a component of exosomes, as some studies showed Tau to be present in exosomes from different cells including neurons [26, 48], whereas others did not find this [11, 12, 49]. Here, we show that Tau is indeed released via exosomes by cultured neurons, by N2a cells overexpressing human Tau and in vivo in human brain. The secretion of exosomal Tau by neurons is not an artifact due to the association with the exosomal surface by Tau protein from dead cells, because we find that Tau is located inside exosomes (Fig. 3). In addition, chemical stimulation of neurons increases secretion of exosomes containing Tau without obvious neurotoxicity (Fig. 2). This further argues that exosomal Tau release is a physiological process regulated by neuronal activity. The failure of some earlier studies to detect exosomal Tau may be explained as follows: (1) Only a small amount of exosomes isolated from limited starting material was used to detect Tau. Since Tau is not a major component of exosomes (Fig. 1a), and since only a small fraction of released Tau (~2%) is associated with exosomes (Fig. 1b), a robust amount of exosomes is necessary for the analysis of associated Tau. (2) Some cell types could encapsulate Tau into exosomes only poorly, as both biosynthesis of exosomes and their contents may vary between cells [32]. It is worth noting that although we detect Tau in exosomes from mature neurons (DIV14-21), we found no Tau in exosomes from immature neurons (DIV9), consistent with another study [29]. Thus, the Tau content of neuronal exosomes appears to be developmentally regulated and cell-type dependent.

We found that the Tau physiologically released via exosomes by cultured neurons was both intact and hypophosphorylated, compared with cytosolic Tau. It is not clear whether exosomes accumulate preferentially Tau in a low state of phosphorylation, or whether the ingested Tau is dephosphorylated by some exosomal phosphatase. It is worth noting that hypophosphorylated intact Tau was detected in conditioned medium of neuronal cultures in a previous study [11], although such extracellular Tau was demonstrated to be non-exosome associated, as Tau is barely detected in exosomal preparations, probably due to the limited material loaded. By contrast, another report stated that only C-terminally truncated Tau lacking the microtubule binding domain is released by cultured neurons or other types of cells [50] and a small fraction of extracellular Tau (<0.2%) is indeed present in exosomes. The cause of the discrepancy (truncated Tau v.s. intact Tau) between these papers is unknown. In addition, there has been a debate whether human CSF contains intact Tau or truncated Tau, but independently of that we observed intact Tau in CSF from AD and control subjects and also in exosomes derived from such CSF (Fig. 8a, c).

Pathological proteins or miRNAs in exosomes have emerged as potential biomarkers for AD and other neurodegenerative diseases [34, 51, 52]. A recent study demonstrated the presence of Tau in exosomes isolated from blood of patients with AD or frontotemporal dementia (FTD) and that the exosomal levels of total Tau and Tau phosphorylated at T181 or S396 for AD and FTD were significantly higher than in control subjects [51]. We show here that Tau is present in exosomes from human CSF as well. However, distinct from blood-derived exosomes, there is no significant difference in the levels of Tau phosphorylated at 12E8 (pS262 + pS356) or PHF1 sites (pS396 + pS404) between CSF-derived exosomes from AD and control cases. In addition, the presence of Tau phosphorylated at 12E8 and PHF1 sites in CSF-derived exosomes is in contrast to the absence of such tau species in exosomes derived from cultured neurons.

Since Tau oligomers are considered the major toxic species in AD, it is of interest to know whether they occur in exosomes as well. We find that SDS-stable Tau oligomers were preferentially encapsulated into CSF-derived exosomes from AD and control cases (Fig. 8a). Consistent with our results, Saman et al. reported the identification of Tau oligomers in exosomes isolated from CSF of AD, but whether Tau oligomers are also present in exosomes from control subjects was not determined in that study [28]. They found that the AD CSF-derived exosomes contained 35-40kD truncated Tau species and Tau trimers of ~105kD [28]. This is different from what we identified here: the exosomal Tau from both AD and control CSFs consisted mainly of species of 55-68kD that likely represent intact Tau isoforms and Tau oligomers of ~180kD that might be trimers of intact Tau (Fig. 8a, c). The cause of the discrepancy between these two studies is unclear, but it may be related to the different preparations of CSF, as Saman et al. used postmortem CSF while we used freshly collected CSF. As Tau oligomers may be neurotoxic, the exosome-mediated release of Tau oligomers may actually be a protective process for neurons to clear these toxins, by analogy to the observation of exosome-mediated release of alpha-synuclein [53]. However, on the other hand, we showed that such exosomes from human CSF promote tau aggregation (Fig. 8e), probably because the exosomal Tau oligomers can seed the aggregation of other Tau molecules, and thereby mediate the propagation of tau pathology.

Some recent studies proposed that Tau pathology can spread via a trans-synaptic pattern [8, 9, 44]. However, direct evidence showing transmission of Tau from presynaptic to postsynaptic compartments is still lacking. Microfluidic devices are excellent tools to study the transitions of Tau between cell compartments and across cells. By using these chambers, we found that Tau is transmitted between neurons via exosomes (Fig. 4). Importantly, we showed that part of the exosomes may be taken up as intact vesicles by the 1^st^ order neurons and then released and internalized by the 2^nd^ order neurons. Our results indicate that the uptake of exosomes does not necessarily mean the fusion of exosomes with the plasma membrane to allow the direct transfer of exosomal content to the cytosol. Consistent with our results it has been shown that exosomes are recruited as single vesicles to the cell cytosol, instead of fusing with plasma membrane, and after internalization, the majority of the internalized exosomes move toward lysosomal compartments [54]. In addition, our results suggest that some internalized exosomes may be spared from this pathway, and can be released and taken up by other neurons.

**Fig. 9:**
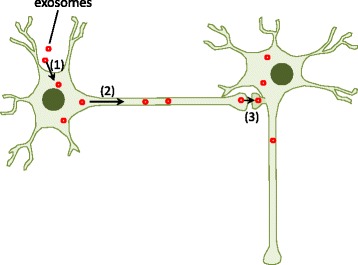
Diagram of exosome-mediated Tau transmission. (1) When Tau-containing exosomes are released into extracellular space, they can be taken up by neurons. (2) The internalized exosomes undergo axonal transport and finally are released at pre-synaptic terminals. (3) The released exosomes are taken up by the synaptically connected neurons, resulting in the distribution of Tau in these recipient cells

Moreover, we showed that the exosome-mediated transmission of Tau^GFP^ requires synaptic connections between the 1^st^ order neurons on the somal side and 2^nd^ order neurons on the neuritic side. This view is based on three observations. (1) No exosomes were detected in conditioned medium on the neuritic side, indicating that the transition of Tau^GFP^ does not occur because exosomes of 1^st^ order neurons are released into the conditioned medium and then internalized by 2^nd^ order neurons at extrasynaptic sites (Fig. 5a, ii). (2) There is no transmission of Tau^GFP^ exosomes from the 1^st^ order to the young 2^nd^ order neurons (DIV4) on the neuritic side (which lack synaptic connections) (Fig. 5c), indicating that synaptic contacts are necessary for exosome-mediated Tau transmission. (3) In the 3-chamber microfluidic devices, transmission of Tau^GFP^ was observed from neurons in the 1^st^ chamber to neurons in the 2^nd^ and 3^rd^ chamber (Fig. 5i). However, no transmission of Tau^GFP^ was observed from neurons in the 1^st^ chamber to neurons in the 3^rd^ chamber, when no neurons were seeded in the 2^nd^ chamber (Additional file 4: Figure S4). In this case, the two populations of neurons do not form synaptic connections, which confirms that synaptic connections are necessary for the transmission of Tau^GFP^. Our results argue that the trans-synaptic transmission of Tau^GFP^ occurs because exosomes are preferentially released at presynapses of the 1^st^ order neurons and subsequently taken up by the postsynapses of the 2^nd^ order neurons (Fig. 9). However, it should be pointed out that here it is the internalized exosomes added to the somal side that are preferentially released at pre-synaptic terminals on the neuritic side. By contrast, the fusion of multivesicular bodies with membranes in cell bodies and dendrites may result in the release of “endogenous” exosomes at these sites as well [39].

Our study showed that Tau aggregates are preferentially released via exosomes by an N2a cell model of Tau aggregation, as higher percentage of Tau is insoluble in exosomes than in N2a cells (Fig. 7a, b). In addition, we found that Tau oligomers are present in human CSF-derived exosomes (Fig. 8a). Importantly, we showed that the Tau-aggregates containing exosomes can induce Tau aggregation in cultured cells (Figs. 7h and 8e). Taken together, our study implies that exosomes could play a role in the spreading of Tau pathology. Notably, exosome-associated Tau aggregates appear to be more efficient in seeding Tau aggregation than the free Tau aggregates, since the broken exosomes failed to induce Tau aggregation. Thus, although exosomal Tau only accounts for a tiny portion of extracellular Tau, the contribution of exosomes in the spreading of Tau pathology may not be negligible. Indeed, this view is supported by a recent study showing that inhibition of exosome synthesis halts tau propagation in a mouse model [55]. In fact, the preferential release of internalized exosomes at pre-synaptic terminals would naturally explain why Tau pathology spreads in a hierarchical pattern rather than in a proximity-dependent pattern. The scenario includes several steps: (1) Tau aggregates containing exosomes are released in one brain area and internalized by neighbouring neurons; (2) Internalized exosomes undergo axonal transport and finally are released only at pre-synaptic terminals. (3) The released exosomes are taken up by the synaptically connected neurons in another area and subsequently induce Tau aggregation in these neurons. Recent studies proposed that certain Tau aggregates can undergo direct trans-synaptic transmission [56, 57]. However, it is not clear whether such Tau aggregates can only be released at pre-synaptic terminals. If such Tau aggregates could be released along the axon shaft, one would expect the occurrence of the proximity-dependent spreading of Tau pathology, unless other mechanisms exist that prevent the uptake of such Tau aggregates by surrounding neurons.

Recently, passive immunotherapies with Tau specific antibodies have been reported to retard the progress of Tau pathology in Tau transgenic mice. It has been proposed that Tau antibodies might achieve therapeutic effects by capturing extracellular Tau seeds and thereby prevent the seeding and trans-cellular propagation of Tau pathology [58–60]. However, given that exosomes shielding the contained Tau aggregates from recognition by antibodies may mediate the propagation of Tau aggregation, there is the question whether antibodies targeting only extracellular Tau can indeed terminate the progression of Tau pathology.

Finally, we found that exosomes are internalized by neurons and microglia but not by astrocytes in cultured organotypic hippocampal slices. Microglia are more efficient than neurons in uptake of exosomes. To date, whether or not extracellular vesicles including exosomes uptake is a cell type-specific process remains a matter of debate [61]. A previous report showed that oligodendrocytes-derived exosomes are predominantly internalized by microglia via micropinocytosis, but not by neurons and astrocytes [62]. The inconsistence between this study and our study may be related to the different origins of exosomes (N2a cells v.s. oligodendrocytes). The significance of the preferential internalization of exosomes by microglia over neurons requires further investigation.

## Conclusion

Our data demonstrate that different Tau species including monomers, oligomers and aggregates may be released via exosomes by neurons or cultured cells. The Tau aggregates-containing exosomes derived from human CSF or cultured cells can promote Tau aggregation. Release of exosomes is enhanced by neuronal activity. Exosomes are involved in the trans-synaptic transmission of Tau between neurons and thus may contribute to the spreading of Tau pathology.

## Additional files

Additional file 1: Figure S1. Tau in exosomes from cortical neurons is not phosphorylated at PHF1 and AT8 sites. Tau in neuronal lysates is phosphorylated at PHF1 and AT8 sites (lanes 1, 4), whereas Tau in dephosphorylated neuronal lysates and exosomal Tau are not detectably phosphorylated at these sites (lane 2, 3 and 5, 6). (PNG 30 kb)
Additional file 2: Figure S2. Atomic force microscopy of exosomes from cortical neurons. (A) The amplitude, height and 3D images of AFM. Scale bar = 100 nm. (B) Quantification of the size of exosomes. The majority of the vesicles are 40–100 nm. (PNG 89 kb)
Additional file 3: Figure S3. Projections of neurons cultured in microfluidic devices with short microgrooves (150 μm). The 1^st^ order hippocampal neurons were seeded on the somal side (left) of the microfluidic chamber. Fourteen days later, the 2^nd^ order hippocampal neurons were seeded on the neuritic side (right) and cultured for additional 10–11 days. Neurons were treated on the neuritic side (A) or somal side (C) with DiI for 2 to 3 h. The living cells were then imaged using fluorescence microscopy. Scale bar = 10 μm. When the 2^nd^ order neurons on the neuritic side were treated with DiI (A, top right panel), cell bodies of the 1^st^ order neurons whose neurites projected through microgrooves to the neuritic side were positive for DiI staining (arrowhead in A, top left panel), by contrast, cell bodies of neurons that do not project to the neuritic side were not positive for DiI staining (arrows in A, bottom left panel). When the 1^st^ order neurons on the somal side were treated with DiI (C, top left panel), their processes that projected through microgrooves to the neuritic side were stained by DiI (C, top right panel). However, no cell bodies of the 2^nd^ order neurons (arrows in C, bottom right) were positive for DiI staining, indicating that the 2^nd^ order neurons do not project through microgrooves to the somal side. The result of A and C is illustrated by B and D resp.. The flow of the conditioned medium (indicated by arrow) prevents the diffusion of added Dil from treated side to the opposite side. (PNG 175 kb)
Additional file 4: Figure S4. No transmission of Tau^GFP^ exosomes from 1^st^ order neurons in the first chamber to the 2^nd^ order neurons in the third chamber. (A) Diagram to show the culture of neurons in 3-chamber microfluidic devices. Neurons were seeded in the 1^st^ and 3^rd^ chambers at the same time, but not in the 2^nd^ chamber. (B) No transmission of Tau^GFP^ exosomes from neurons (DIV10) cultured in 1^st^ chamber to neurons cultured in 3^rd^ chamber. The 1^st^ order neurons cultured in the 1^st^ chamber were treated with Tau^GFP^ exosomes (20 μg) at DIV10 for 24 h. Neurons were stained with an antibody against tubulin (red). Arrows indicate Tau^GFP^ positive exosomes in the first channel and in the axons projecting from the 1^st^ order neurons. Note the absence of Tau^GFP^ positive exosomes in the third channel indicating no transmission of Tau^GFP^ via exosomes between the two populations of neurons. Scale bar = 10 μm. (PNG 118 kb)
Additional file 5: Figure S5. Uptake and transmission of Tau^CFP^ by neurons cultured in microfluidic chambers via exosomes derived from cultured neurons. (A) Nanoparticle tracking analysis of exosomes derived from primary neurons transfected with Tau^CFP^. The size distribution peaks at ~90 nm, indicating the enrichment of exosomes in the preparations. (B) Uptake and transmission of exosomes containing Tau^CFP^ isolated as in (A) by neurons cultured in microfluidic chambers with long microgrooves (900 nm). The 1^st^ order neurons at DIV25 were treated for 24 h with exosomes (20 μg) isolated from primary cortical neurons infected with adeno-virus expressing Tau^CFP^, when the 2^nd^ order neurons were at DIV11. Neurons were then fixed and stained for immunofluorescence microscopy with antibodies against MAP2 (red). Arrows denote Tau^CFP^ exosomes. Scale bar = 10 μm. Note that Tau^CFP^ exosomes were detected in the 2^nd^ order neurons on the neuritic side, indicating their uptake by 1^st^ order neurons on the somal side, transport across the microgrooves, and synaptic transmission to the neurons on the neuritic side. (PNG 157 kb)
Additional file 6: Figure S6. No uptake of sonicated exosomes derived from N2a cells expressing Tau^GFP^ by neurons or astrocytes in organotypic hippocampal slices. Hippocampal slice cultures were incubated for 24 h with sonicated exosomes (20 μg) derived from N2a cells expressing Tau^GFP^. Cultures were then stained for neuronal marker MAP2 (red) and astrocytic marker GFAP (blue) in hippocampal CA1 region. No Tau^GFP^ was observed in slices. Scale bars in upper panel = 20 μm; in bottom panel = 5 μm. (PNG 191 kb)
Additional file 7: Figure S7. Nanoparticle tracking analysis of exosomes isolated from CSF of control (A) and AD subjects (B) showing particle number vs. size (arbitrary units, peak = 100%). The size distributions have peaks at diameters of ~85–100 nm (AD) or ~60–90 nm (non-AD controls). (PNG 29 kb)

## Abbreviations

AD: Alzheimer Disease
AFM: Atomic force microscopy
BSA: Bovine serum albumin
CSF: Cerebrospinal fluid
DIV: Days in vitro
EVs: Extracellular vesicles
FBS: Fetal bovine serum
FTD: Frontotemporal dementia
FTDP-17: Familial frontotemporal dementia and parkinsonism linked to chromosome17
HSC70: Heat Shock Cognate Protein 70
LDH: Lactate dehydrogenase
miRNA: MicroRNA
NFTs: Neurofibrillary tangles
NMJ: Neuromuscular junctions
NTA: Nanoparticle tracking analysis
Tau^GFP^: Human longest isoform of brain Tau tagged with GFP at the N-terminus
Tau^RDΔK^: Four-repeat domain of Tau with the mutation ΔK280
TEM: Transmission electron microscopy
