# Supplementary figures and images for: The release and trans-synaptic transmission of Tau via exosomes
Source: Mol Neurodegener. 2017 Jan 13;12:5. doi: 10.1186/s13024-016-0143-y (PMC5237256; doi:10.1186/s13024-016-0143-y)

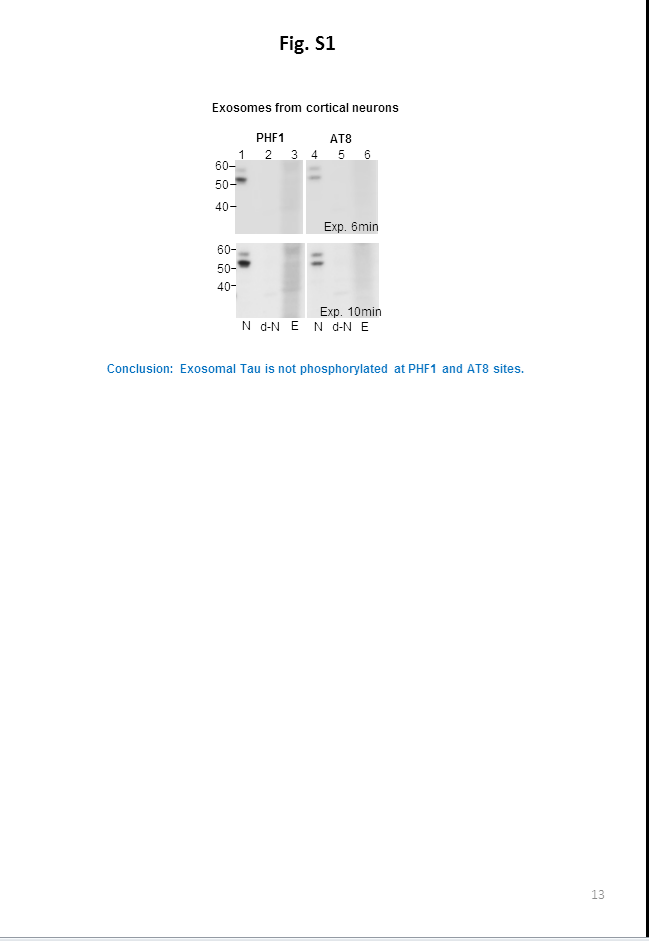

Supplement: Additional file 1: Figure S1. — Tau in exosomes from cortical neurons is not phosphorylated at PHF1 and AT8 sites. Tau in neuronal lysates is phosphorylated at PHF1 and AT8 sites (lanes 1, 4), whereas Tau in dephosphorylated neuronal lysates and exosomal Tau are not detectably phosphorylated at these sites (lane 2, 3 and 5, 6). (PNG 30 kb) [file 13024_2016_143_MOESM1_ESM.png]

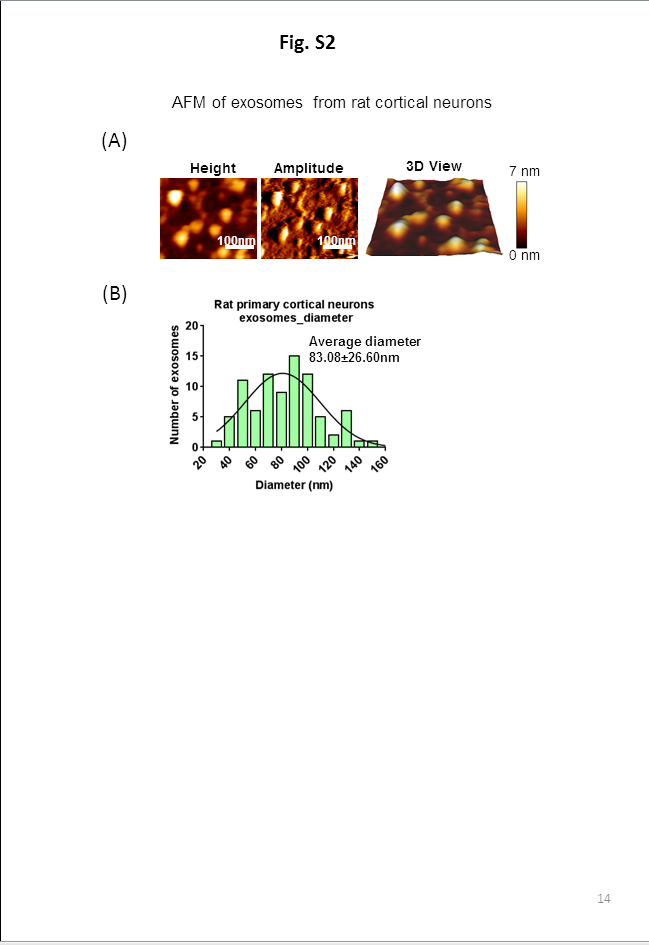

Supplement: Additional file 2: Figure S2. — Atomic force microscopy of exosomes from cortical neurons. (A) The amplitude, height and 3D images of AFM. Scale bar = 100 nm. (B) Quantification of the size of exosomes. The majority of the vesicles are 40–100 nm. (PNG 89 kb) [file 13024_2016_143_MOESM2_ESM.png]

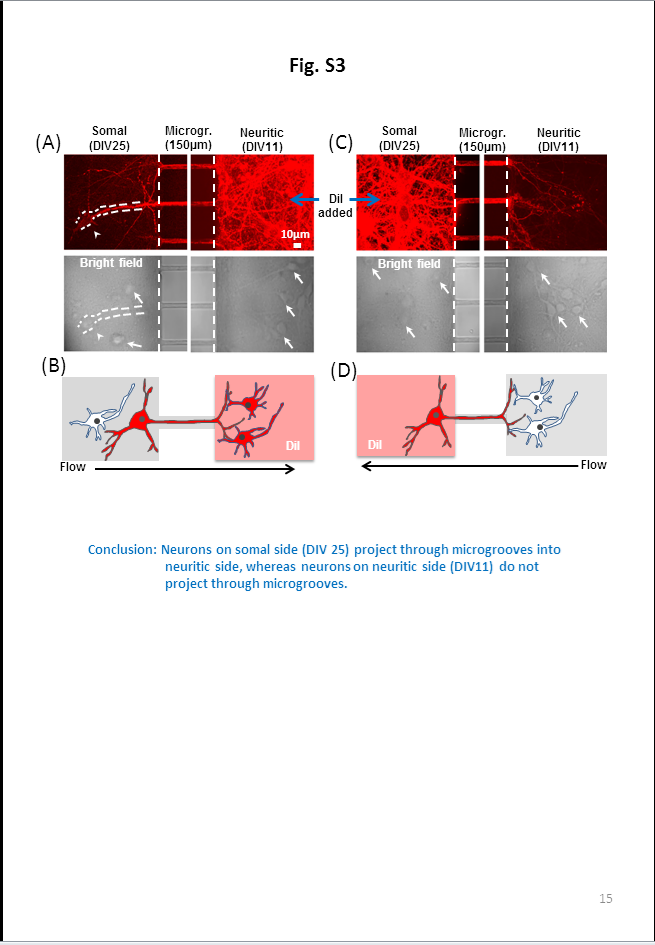

Supplement: Additional file 3: Figure S3. — Projections of neurons cultured in microfluidic devices with short microgrooves (150 μm). The 1st order hippocampal neurons were seeded on the somal side (left) of the microfluidic chamber. Fourteen days later, the 2nd order hippocampal neurons were seeded on the neuritic side (right) and cultured for additional 10–11 days. Neurons were treated on the neuritic side (A) or somal side (C) with DiI for 2 to 3 h. The living cells were then imaged using fluorescence microscopy. Scale bar = 10 μm. When the 2nd order neurons on the neuritic side were treated with DiI (A, top right panel), cell bodies of the 1st order neurons whose neurites projected through microgrooves to the neuritic side were positive for DiI staining (arrowhead in A, top left panel), by contrast, cell bodies of neurons that do not project to the neuritic side were not positive for DiI staining (arrows in A, bottom left panel). When the 1st order neurons on the somal side were treated with DiI (C, top left panel), their processes that projected through microgrooves to the neuritic side were stained by DiI (C, top right panel). However, no cell bodies of the 2nd order neurons (arrows in C, bottom right) were positive for DiI staining, indicating that the 2nd order neurons do not project through microgrooves to the somal side. The result of A and C is illustrated by B and D resp.. The flow of the conditioned medium (indicated by arrow) prevents the diffusion of added Dil from treated side to the opposite side. (PNG 175 kb) [file 13024_2016_143_MOESM3_ESM.png]

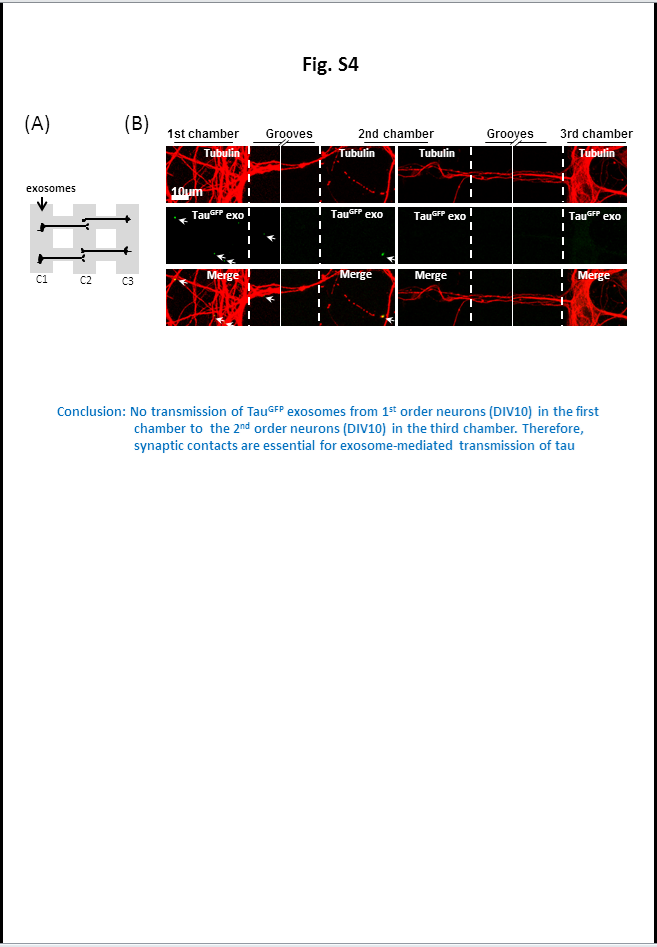

Supplement: Additional file 4: Figure S4. — No transmission of TauGFP exosomes from 1st order neurons in the first chamber to the 2nd order neurons in the third chamber. (A) Diagram to show the culture of neurons in 3-chamber microfluidic devices. Neurons were seeded in the 1st and 3rd chambers at the same time, but not in the 2nd chamber. (B) No transmission of TauGFP exosomes from neurons (DIV10) cultured in 1st chamber to neurons cultured in 3rd chamber. The 1st order neurons cultured in the 1st chamber were treated with TauGFP exosomes (20 μg) at DIV10 for 24 h. Neurons were stained with an antibody against tubulin (red). Arrows indicate TauGFP positive exosomes in the first channel and in the axons projecting from the 1st order neurons. Note the absence of TauGFP positive exosomes in the third channel indicating no transmission of TauGFP via exosomes between the two populations of neurons. Scale bar = 10 μm. (PNG 118 kb) [file 13024_2016_143_MOESM4_ESM.png]

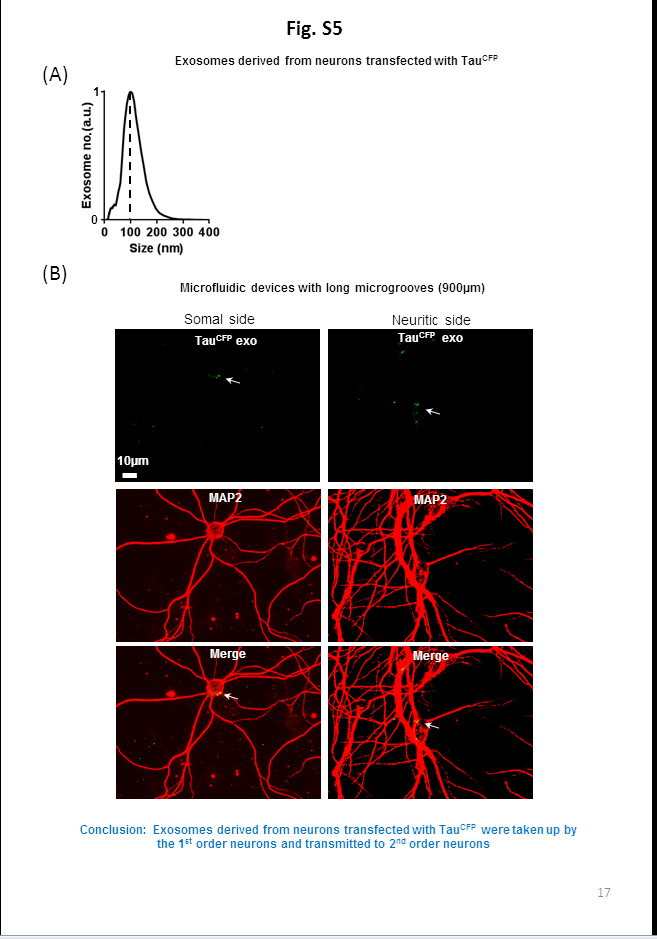

Supplement: Additional file 5: Figure S5. — Uptake and transmission of TauCFP by neurons cultured in microfluidic chambers via exosomes derived from cultured neurons. (A) Nanoparticle tracking analysis of exosomes derived from primary neurons transfected with TauCFP. The size distribution peaks at ~90 nm, indicating the enrichment of exosomes in the preparations. (B) Uptake and transmission of exosomes containing TauCFP isolated as in (A) by neurons cultured in microfluidic chambers with long microgrooves (900 nm). The 1st order neurons at DIV25 were treated for 24 h with exosomes (20 μg) isolated from primary cortical neurons infected with adeno-virus expressing TauCFP, when the 2nd order neurons were at DIV11. Neurons were then fixed and stained for immunofluorescence microscopy with antibodies against MAP2 (red). Arrows denote TauCFP exosomes. Scale bar = 10 μm. Note that TauCFP exosomes were detected in the 2nd order neurons on the neuritic side, indicating their uptake by 1st order neurons on the somal side, transport across the microgrooves, and synaptic transmission to the neurons on the neuritic side. (PNG 157 kb) [file 13024_2016_143_MOESM5_ESM.png]

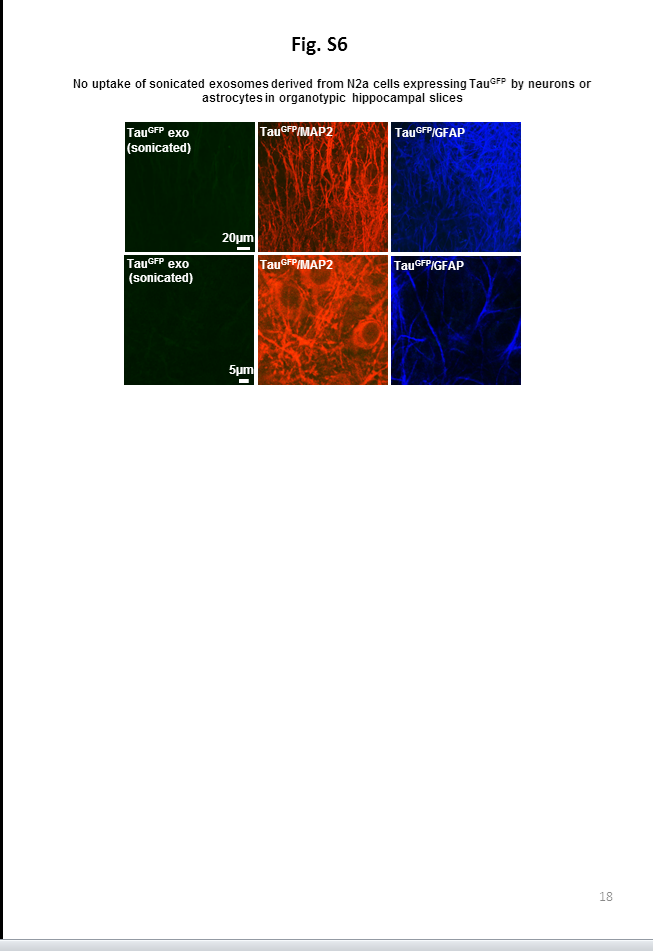

Supplement: Additional file 6: Figure S6. — No uptake of sonicated exosomes derived from N2a cells expressing TauGFP by neurons or astrocytes in organotypic hippocampal slices. Hippocampal slice cultures were incubated for 24 h with sonicated exosomes (20 μg) derived from N2a cells expressing TauGFP. Cultures were then stained for neuronal marker MAP2 (red) and astrocytic marker GFAP (blue) in hippocampal CA1 region. No TauGFP was observed in slices. Scale bars in upper panel = 20 μm; in bottom panel = 5 μm. (PNG 191 kb) [file 13024_2016_143_MOESM6_ESM.png]

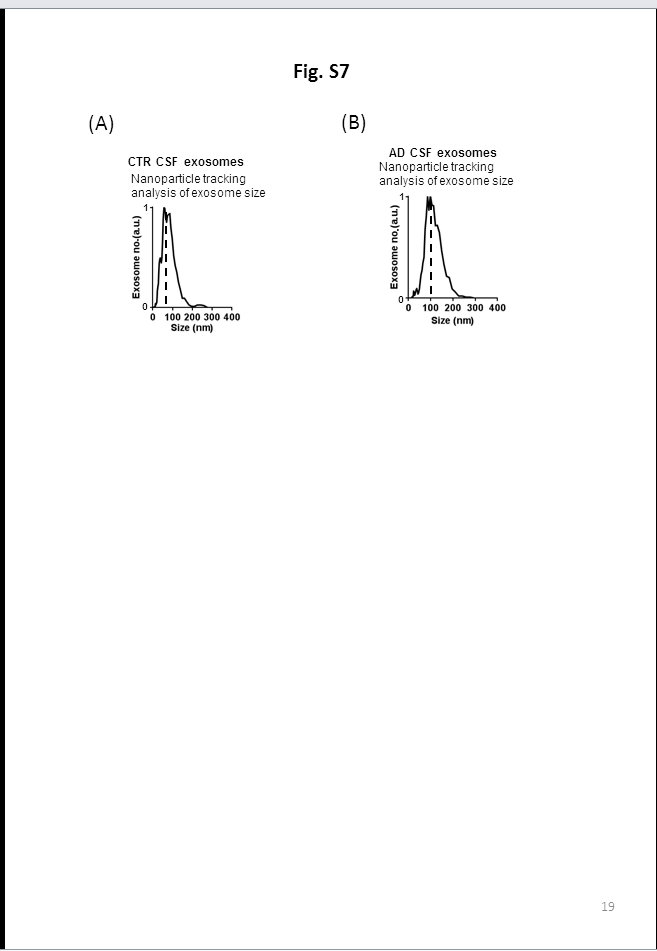

Supplement: Additional file 7: Figure S7. — Nanoparticle tracking analysis of exosomes isolated from CSF of control (A) and AD subjects (B) showing particle number vs. size (arbitrary units, peak = 100%). The size distributions have peaks at diameters of ~85–100 nm (AD) or ~60–90 nm (non-AD controls). (PNG 29 kb) [file 13024_2016_143_MOESM7_ESM.png]
